# Functional and Nutritional Potential of Chickpea Protein Hydrolysates: A Systematic Review and Plant-protein Network Analysis

**DOI:** 10.1007/s11130-026-01568-z

**Published:** 2026-09-02

**Authors:** Noelia M. Rodríguez-Martín, José Carlos Márquez-López, María-Soledad Fernández-Pachón, Justo Pedroche

**Affiliations:** 1 Department of Biological and Health Sciences, Faculty of Health Sciences, Loyola University (Universidad Loyola Andalucía), Avda. de las Universidades s/n, Dos Hermanas, 41704 Spain; 2 Plant Protein Group, Instituto de la Grasa-CSIC, Avda. Rectora Rosario Valpuesta nº 1, Dos Hermanas Seville, 41089 Spain; 3 Área de Nutrición y Bromatología, Departamento de Biología Molecular e Ingeniería Bioquímica, Universidad Pablo de Olavide, Avda. Rectora Rosario Valpuesta nº 1, Dos Hermanas Seville, 41089 Spain

**Keywords:** *Cicer arietinum*, Enzymatic hydrolysis, Bioactive properties, Simulated gastrointestinal digestion, Preclinical evidence, Bibliometric mapping, Plant-based ingredients

## Abstract

Chickpea is a protein-rich legume increasingly explored as a substrate for functional plant-based ingredients. Chickpea protein hydrolysates (CPHs) and chickpea-derived peptides (CPs), obtained through enzymatic hydrolysis or simulated gastrointestinal digestion, may provide technological and biological properties while supporting the valorization of chickpea fractions and by-products. This review integrates a network analysis of title–abstract terms from 5,728 unique Scopus and PubMed records on plant protein hydrolysates with a systematic review of 72 studies focused on CPH production, peptide characterization, bioactivity, and translational gaps. The evidence indicates that CPHs and CPs show promising antioxidant, antihypertensive, antidiabetic, anti-inflammatory, lipid-lowering, immunomodulatory, antimicrobial, and anticancer-related activities, mainly supported by biochemical assays, cell models, and animal studies. However, heterogeneous hydrolysis protocols, incomplete peptide characterization, inconsistent bioactivity methods, limited scale-up evidence, and the absence of human intervention trials restrict translation. Future studies should prioritize standardized protocols, mechanistic validation, bioavailability, sensory and regulatory assessment, food-matrix validation, and clinical trials.

**Supplementary Information:**

The online version contains supplementary material available at https://doi.org/10.1007/s11130-026-01568-z.

## Introduction

Chickpea (*Cicer arietinum* L.) is one of the most widely cultivated and consumed pulses worldwide and represents an important component of Mediterranean, Middle Eastern, and South Asian diets. Its nutritional value, adaptability to dry conditions, nitrogen-fixing capacity, and suitability for food innovation make chickpea a relevant crop within sustainable plant-based food systems [1–3].

Chickpea processing operations, including cleaning, grading, dehulling, and milling, generate chickpea-derived by-products such as seed coats, broken seeds, and undersized or damaged grains. While traditionally destined for animal feed, their nutritional potential makes them suitable for upcycling into novel foods, thus contributing to circular economy strategies [1, 2]. Chickpea flour has been widely incorporated into baked goods, snacks, extruded and gluten-free products, and plant-based beverages [4, 5]. Moreover, chickpea-derived by-products could also be incorporated into novel food formulations. For example, non-compliant chickpea and pea residues have been used to fortify sourdough bread [RS1], improving their nutritional composition.

Beyond direct food uses, proteolysis is extensively investigated as an approach to generate protein hydrolysates and bioactive peptides, although most applications remain at the laboratory or pilot scale. Protein hydrolysis may improve technological properties such as solubility and emulsification and reduce viscosity [1, 6], while potentially decreasing allergenicity and enhancing digestibility and bioavailability [7]. However, broader industrial implementation remains constrained by production costs, scalability, and sensory limitations such as bitterness. Over the last 30 years numerous bioactivities have been attributed to protein hydrolysates and peptides, including antihypertensive, anti-inflammatory, lipid-lowering, among other effects [8].

Chickpea protein hydrolysates (CPHs) and peptides (CPs) may therefore be of interest for formulation into functional foods and nutritional supplements, including infant formulas, sports products, enteral diets, and specialized nutrition products [6, 7, 9].

This review builds on current evidence to (i) examine research trends in plant protein hydrolysates through network analysis and establish the broader research landscape; (ii) position chickpea within this landscape and contextualize its potential as a protein-rich matrix for the production of bioactive hydrolysates and peptides; (iii) systematically evaluate the production methods, hydrolysis conditions, peptide characterization, and bioactivity assessment of CPHs and CPs; and (iv) identify the main translational gaps affecting their development as functional plant-based ingredients.

## Procedure and Data Analysis

### Network Analysis

A network analysis was conducted using records retrieved from Scopus and PubMed on August 24, 2026, covering publications from 1942 to 2026. The search strategy was: “protein AND hydrolysate AND (seed OR cereal OR legume OR lentil OR quinoa OR chia OR chickpea OR soy OR bean OR rice OR wheat OR maize OR teff OR hemp)”. The search yielded 4,409 records from Scopus and 3,179 from PubMed. After deduplication based on DOI and PMID using the dplyr package in R, 5,728 unique records were retained. Of these, 110 (1.92%) explicitly mentioned chickpea in their titles, indicating that it represents a relatively small segment of the broader plant protein hydrolysate literature.

VOSviewer was used to construct a title–abstract term co-occurrence network using binary counting. The analysis mapped relationships among plant sources, hydrolytic enzymes, physicochemical and techno-functional properties, bioactivities, and experimental models, thereby positioning chickpea within the broader research landscape and providing context for the subsequent systematic synthesis of CPHs. A thesaurus was used to merge synonyms and near-duplicate terms and to exclude general or uninformative vocabulary, measurement units, non-plant protein hydrolysates, and unrelated topics. Following manual relevance screening, 96 connected terms were retained to produce a focused and interpretable representation aligned with the objectives of the review.

### Systematic Review

Following PRISMA 2020 (Online Resource Table S1), a systematic review evaluated CPH and CP bioactivity. The 110 chickpea-related records from the bibliometric analysis formed the initial pool. Because that search mapped the broader plant-protein-hydrolysate field, a focused PubMed and Scopus sensitivity search (“protein AND hydrolysate AND chickpea”) was conducted on August 24, 2026 to ensure chickpea-specific coverage. Eligible studies experimentally assessed any CPH bioactivity using biochemical, cell-based, or animal assays, or purified or identified CPs linked to these outcomes. Reviews, studies focused only on chemical characterization, technological properties, allergy, peptides whose chickpea origin was unclear, or mixtures with animal protein hydrolysates were excluded.

**Fig. 1:**
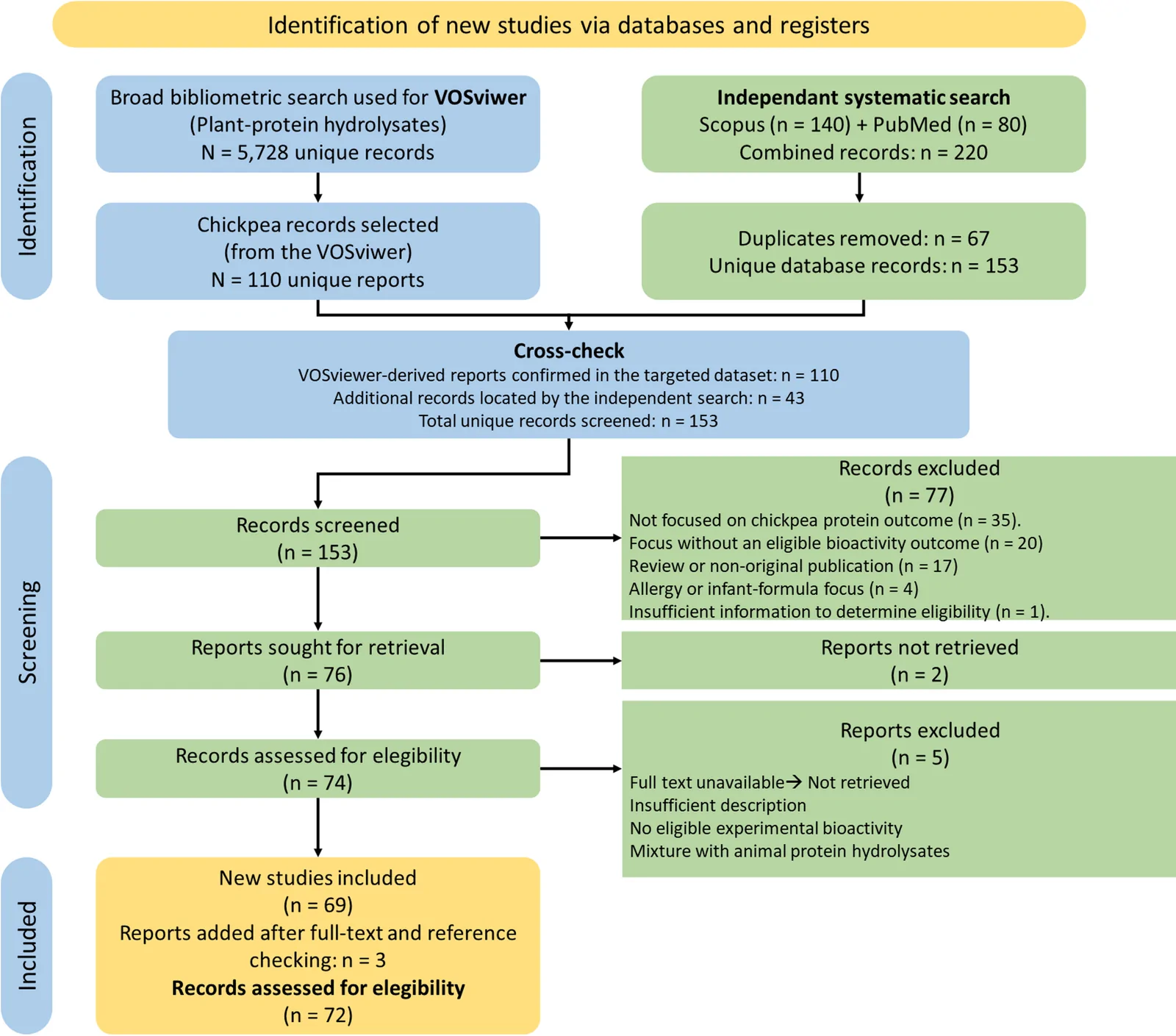
PRISMA-based flowchart. The flowchart illustrates the study selection process for the systematic review, including identification from PubMed and Scopus, duplicate removal, title and abstract screening, full-text eligibility assessment, and final inclusion of 72 studies

Article selection was performed independently by two authors (N.M.R.-M. and J.C.M.-L.) based on titles, abstracts, keywords, and full-text assessment, with disagreements resolved by consensus. The initial search identified 80 records in PubMed and 140 in Scopus. After screening and full-text evaluation, 72 studies were included in the systematic review. Of the 72 studies included in the final systematic review, 69 were contained within the bibliometric corpus, whereas three additional studies were identified through backward citation searching of relevant reference lists. The study selection process is shown in Fig. 1, and the detailed data extraction, including substrate, pretreatment, hydrolysis conditions, peptide characterization, bioactivity assays, and biological models, is provided in Online Resource Tables S2–S3 and Fig. S1-S2.

## Chickpea as a Matrix for Bioactive Protein Hydrolysates

Chickpeas contain approximately 62–70% carbohydrates, 15–25% protein, 15–22% dietary fibre, and 4–6% fat, although these values may vary depending on genotype, growing conditions, and processing [6, 7]. A detailed summary of chickpea seed composition, including proximate composition, micronutrients, fatty acids, and amino acid profile, is provided in Online Resource Tables S4–S7 to support the description of chickpea as a protein-rich matrix for hydrolysate production.

In the context of this review, although carbohydrates represent the major macronutrient fraction, chickpea is mainly considered a protein-rich matrix for the generation of bioactive hydrolysates and peptides. Chickpea proteins are composed of globulin (salt-soluble proteins), glutelin (soluble in chaotropic/reducing agents), albumin (water-soluble proteins), and prolamin (alcohol-soluble proteins), and representing 53–60%, 19–25%, 8–12%, and 3–7%, of total protein, respectively [6]. Chickpea proteins also provide a favourable amino acid profile, with relevant amounts of lysine (49–72 mg/g), arginine (83–98 mg/g), glutamic acid (131–187 mg/g), and hydrophobic residues, which may contribute to the generation of peptides with functional properties.

Chickpea processing generates protein-containing by-products and downgraded fractions, including broken, undersized, or damaged grains, which are often underused despite their potential as substrates for value-added food ingredients.

The use of food-grade proteases enables the production of CPHs with improved solubility, reduced viscosity, enhanced dispersion, and potentially better incorporation into food matrices [6]. Beyond these technological advantages, enzymatic hydrolysis can release low-molecular-weight peptides with biological activity, commonly referred to as bioactive peptides [10]. Therefore, protein hydrolysis represents a promising strategy to transform chickpea proteins and protein-rich by-products into functional plant-based ingredients while contributing to waste reduction.

## Current Evidence on Chickpea Protein Hydrolysates

The following sections synthesize the evidence obtained from the network analysis and systematic review, focusing on research trends, production conditions, peptide characterization, bioactivity assessment, and translational gaps.

### Network Analysis: Research Trends and Applications

**Fig. 2:**
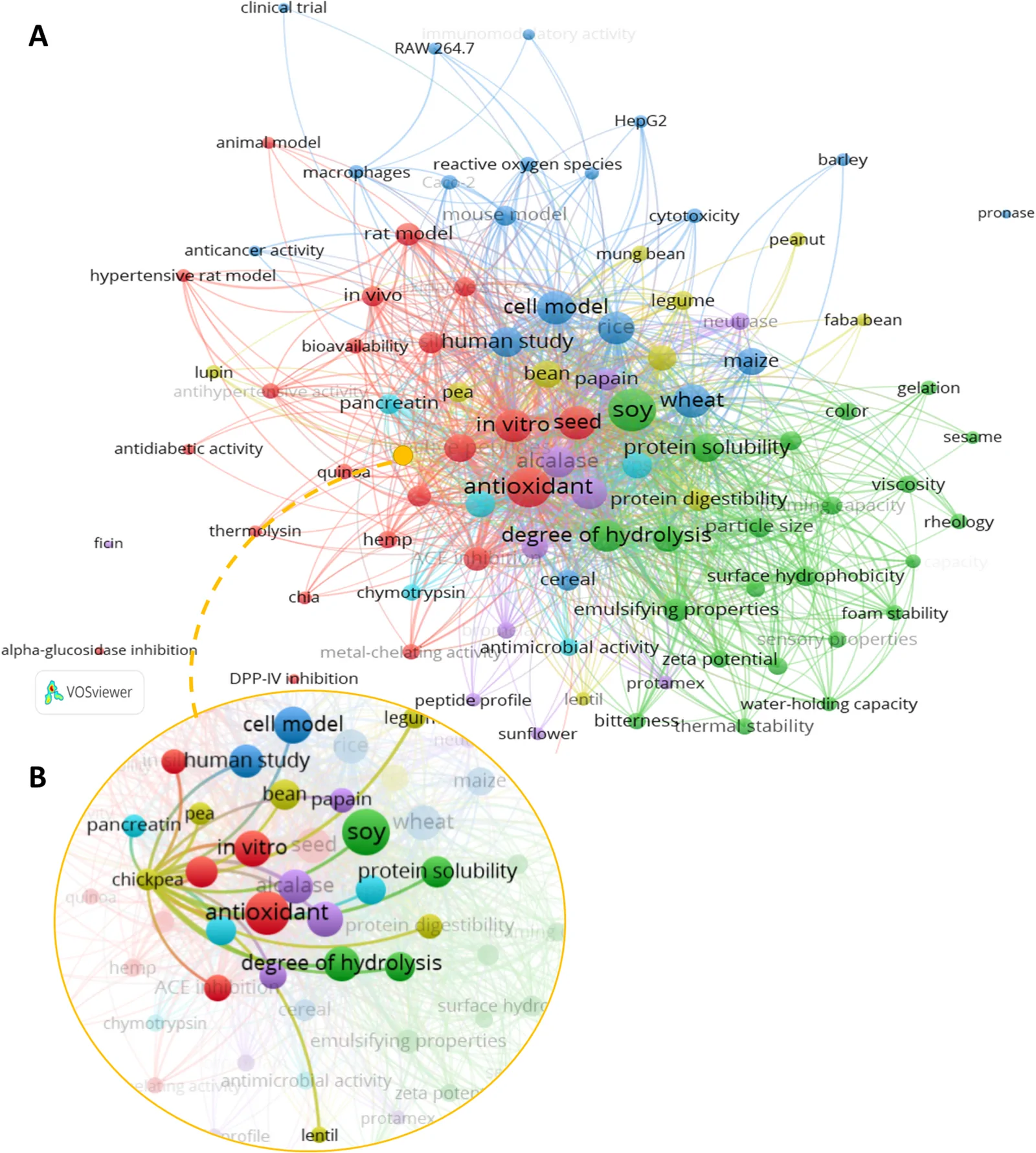
Title–abstract term co-occurrence network of 5,728 plant protein hydrolysate records published between 1942 and 2026, generated using VOSviewer. (**A**) Complete network of selected terms. (**B**) Same network highlighting chickpea and its connections. Node size indicates frequency, colors represent clusters, and line thickness reflects co-occurrence strength

The network analysis first provided a broader perspective on the plant-protein-hydrolysate research landscape. The title–abstract term co-occurrence network comprised 96 selected terms distributed across six clusters (Fig. 2). Among the most prominent terms were ‘soy’, ‘antioxidant’, ‘cell model’, ‘in vitro’, ‘protease’, ‘degree of hydrolysis’, and ‘alcalase’. The principal research areas encompassed enzymatic hydrolysis, physicochemical and techno-functional properties, antioxidant and enzyme-inhibitory activities, gastrointestinal digestion, and cellular, animal, and human models.

Within this broader landscape, chickpea was particularly associated with antioxidant activity, Alcalase, degree of hydrolysis, in vitro assays, pepsin, protein solubility, bioactive peptides, Flavourzyme and ACE inhibition (Fig. 2B). These relationships provide the contextual basis for the following focused synthesis of CPHs. The complete term and cluster information is provided in Online Resources M1.

### Systematic Review: Production Conditions and Bioactive Potential

#### Raw Material and Pretreatment Conditions

Among the 72 selected studies, both Kabuli- and Desi-type chickpeas were used, although only 15 studies specifically reported the genotype, including examples such as ICC3761 or Blanco Sinaloa 92. The remaining studies used commercial chickpea seeds, flours, protein isolates, or protein concentrates. Although genotype may influence protein composition and functionality, the variability observed among CPHs appears to be more strongly driven by processing conditions, enzyme specificity, hydrolysis parameters, and experimental design, as detailed in Online Resource Tables S2–S3 and Fig. S1.

Most studies applied at least one pretreatment before hydrolysis to modify the protein matrix and promote peptide release. These pretreatments included germination or sprouting in 7 studies [11–17], precooking or preheating (at 80 °C for 5 to 30 min) in 10 articles [18–27], cooking in 6 manuscripts [14, 18, 22, 28–30], high hydrostatic pressure treatment or autoclaving in 3 studies [28, 31, 32], extrusion in one [33], and ultrasound treatment [RS2, RS3, RS4]. Defatting was also frequently performed, mainly using organic solvents. Since pretreatment protocols remain highly heterogeneous, direct comparison and scale-up are still limited.

### Protein Isolation and Hydrolysis Conditions

Protein isolation was most commonly performed using a two-step procedure, reported in 37 studies, based on alkaline solubilization, generally at pH 8–12, followed by isoelectric precipitation at pH 3.5–5.5 [18, 19, 22, 23, 30, 34–37], [RS5, RS6]. Other studies isolated specific protein fractions using Osborne-type fractionation or albumin- and/or globulin-enriched fractions, indicating that substrate selection may influence the peptide profile generated during hydrolysis [38–41], [RS7]. In addition, one in silico study simulated hydrolysis directly from chickpea legumin and provicilin sequences [42].

Hydrolysis conditions varied widely across studies, particularly regarding enzyme type, pH, temperature, hydrolysis time, enzyme/substrate ratio, and degree of hydrolysis (DH). Alcalase was the most frequently used enzyme, appearing in more than 50% of the reviewed studies, in agreement with the network analysis [21–23, 30, 31, 34–37, 43], [RS2, RS5]. Other enzymes included pancreatin, pepsin, papain, trypsin, bromelain, Flavourzyme, ficin, Neutrase, Protamex, Bioprotease, chymotrypsin, and Protoferm [12, 17–19, 27, 28, 37, 44–46], [RS8]. Several studies also used sequential hydrolysis protocols, including Alcalase–Flavourzyme, pepsin–pancreatin, pepsin–trypsin, or pepsin–trypsin–chymotrypsin combinations, some of which were associated with simulated gastrointestinal digestion (SGID) [22, 35–37, 44, 45, 47], [RS7]. Overall, substrate type, enzyme specificity, hydrolysis time, DH, and pretreatment conditions are key determinants of peptide size distribution, functionality, bioactivity, and reproducibility.

### Methods for Simulated Gastrointestinal Digestion (SGID)

SGID approaches were used in 20 reviewed studies to assess protein digestibility, peptide release, peptide resistance to gastrointestinal digestion, and preservation or enhancement of bioactivity. The most frequently used method was the pepsin–pancreatin protocol, reported in nine studies [12, 18, 19, 24, 31, 34, 40, 44, 48], which is based on a two-step gastric and intestinal digestion protocol originally described by Akeson and Stahmann [47]. Other studies used trypsin–chymotrypsin-based digestion [49], pepsin–trypsin–chymotrypsin approaches derived from Vermeirssen et al. [50], or pepsin–Corolase digestion [22].

More recent studies adopted the standardized INFOGEST protocol developed by Brodkorb et al. [51] which was used in 4 studies [27–29, 52], or INFOGEST-like approaches based on oral, gastric, and intestinal phases [14, 29, 33]. Compared with simpler two-step digestion methods, INFOGEST-based protocols provide a more physiologically structured simulation of digestion and are useful for evaluating peptide bioaccessibility and stability under gastrointestinal conditions. Overall, the diversity of SGID protocols limits direct comparison among studies, but these approaches remain essential to determine whether CPH-derived peptides can resist digestion and retain or modify their bioactivity after gastrointestinal exposure. In this sense, only one study reported PDCAAS values using the pepsin–pancreatin method, showing a value of 78.01 ± 3.07 for Bioprotease-derived CPH [34]. Additionally, Gupta and Bhagyawant assessed the in vitro protein digestibility of chickpea flour suspensions using a trypsin–chymotrypsin method and reported values ranging from 75.56 to 87.15% [49].

Several studies also evaluated whether bioactivity was preserved after SGID. Arnal et al. [28] reported that α-amylase inhibitory activity was maintained after INFOGEST digestion in cooked chickpeas, whereas Zan et al. [27] identified chickpea-derived peptides with alcohol dehydrogenase-activating activity following simulated digestion of CPHs. Similar studies using other SGID methods also reported conserved antioxidant activity in cooked chickpeas [29] and the digestates of protein emulsions [22], as well as in CPH-enriched maize tortilla [18] and in optimized CPH [31]. In addition, germinated chickpeas showed higher anti-inflammatory potential after SGID than cooked chickpeas [14], while other studies reported SGID-related changes in bioactivity associated with protein conformational modifications [13] or with endogenous chickpea protein fractions involved in starch digestion kinetics [53]. SGID also allowed the release of potential antidiabetic and antihypertensive peptides which was demonstrated, either by biochemical analyses [12, 18, 19, 44], in vitro assays [49], or in vivo models [24, 33].

Overall, static SGID methods, including INFOGEST, support reproducible screening, whereas more physiologically representative dynamic models remain absent from CPH research, likely because of their complexity and resource demands.

### Bioactive Peptide Isolation and Identification Techniques

Although peptide isolation and sequence identification are essential to link CP structure with bioactivity, purification workflows are costly, time-consuming, and difficult to scale, which limits their direct applicability in food production [54]. For this reason, the use of whole CPHs or peptide-rich fractions may be more realistic for food applications. Despite differences in substrates, enzymes, and processing conditions, several recurring chickpea-derived sequences and motifs have been reported, including FDLPAL and shorter fragments such as DLPAL, LP, PL, PxLP, DF, FD, DL, FxP, LxP, PLxF, and PxL. These repeated motifs, mainly derived from conserved chickpea storage proteins, support the existence of reproducible peptide signatures with potential relevance for bioactivity.

### Bioactivity of CPHs Using Biochemical Assays

#### Metal-Chelating Activities

Metal-chelating activity was evaluated in nine studies, mainly using spectrophotometric assays for iron, copper, or zinc binding [20, 23, 26, 29, 38–40, 43, 55]. In addition, one study used mass spectrometry to identify Zn-binding CP complexes [46].

Several residues, including Asp, Arg, Asn, Ser, Met, Cys, Thr, and His, have been associated with the metal-binding capacity of CPs [38–40, 46, 56]. These findings suggest that chickpea-derived peptides may contribute to antioxidant protection by limiting metal-catalyzed oxidative reactions. They also support additional applications reported in the literature, including mineral delivery through digestible metal-binding peptides and the formation of metal–peptide complexes, such as vanadium–CP complexes, with improved bio-penetrating capacity and potential anticancer relevance [46, 56].

### Antioxidant Capacity

Antioxidant capacity has been widely evaluated in CPH research using spectrophotometric and fluorometric assays based on radical scavenging and reducing power, commonly with BHT or Trolox as reference antioxidants. The most frequent methods were FRAP/reducing power and ABTS assays, each used in nine studies, followed by hydroxyl radical scavenging in seven studies, ORAC in three studies, and DPPH and NO radical scavenging in one study each. Complementary lipid oxidation models, including β-carotene bleaching, TBARS, and linoleic acid autooxidation assays, were also applied to assess the ability of CPHs and CPs to delay or inhibit lipid oxidation [38, 57].

Several processing strategies, including high hydrostatic pressure (20 min at 200 MPa), autoclaving (20 min at 121 °C), preheating or precooking (> 80 °C for 5–20 min), and germination, including selenium-assisted germination with Na₂SeO₃ for 2 days, have been reported to enhance the antioxidant capacity of CPHs or CPs [16, 22, 24, 25, 31, 32].

In addition, protein fractions or CPs with strong metal-chelating activity often showed higher antioxidant potential and greater inhibition of lipid peroxidation, in some cases associated with increased histidine content and the contribution of imidazole groups to metal coordination and radical-scavenging mechanisms [38–40, 55, 57, 58].

These reviewed studies consistently indicate that protein hydrolysis can enhance antioxidant activity compared with native chickpea proteins. However, direct comparisons among studies remain limited by heterogeneous units and endpoints, including percentage inhibition, IC50 values, Trolox equivalents, MDA equivalents, and assay-specific absorbance or fluorescence readouts.

### Enzyme Inhibition Activity

Enzyme inhibition assays were mainly used to evaluate antihypertensive, antidiabetic, lipid-related, and other metabolic activities of CPHs and CPs. ACE inhibition was the most frequent approach, reported in 13 studies, with IC50 values generally ranging from 0.140 to 0.673 mg/mL, although higher values were also reported depending on substrate, enzyme, and pretreatment conditions [12, 22, 28, 30, 35, 36, 42, 46, 49, 59–62]. DPP-IV inhibition was evaluated in seven studies, with reported IC50 values from 0.17 to 3.05 mg/mL [12, 18, 19, 22, 28, 44, 61], while α-amylase and α-glucosidase inhibition were assessed in four and three studies, respectively [19, 25, 44, 53]. Values of 38.4 ± 1.4% inhibition for SGID hydrolysates and 11.0 ± 0.8% for bromelain hydrolysates at 10 mg/mL were reported. The inhibition of α-Glucosidase was also evaluated in 3 studies, ranging from 53 to 63% [19, 25, 44]. Less frequently explored targets included alcohol dehydrogenase, HMG-CoA reductase, and cholesterol micellar solubility, suggesting additional but still preliminary metabolic applications [9, 27, 31] Overall, these assays support the bioactive potential of CPHs and CPs, although cross-study comparison remains limited by heterogeneous substrates, hydrolysis conditions, and assay protocols.

### Antifungal and Antibacterial Activities

Studies based on antifungal and antibacterial activities are often used to evaluate the potential of ingredients as food preservatives. These activities are typically assessed through assays on bacterial and fungal strains. Heymich et al. [63, 64] identified two CPs, named Leg1 (RIKTVTSFDLPALRFLKL) and Leg2 (RIKTVTSFDLPALRWLKL), which showed potential as preservatives in food and other products by preventing bacterial and fungal spoilage as well as oxidative degradation [63–65].

Furthermore, two smaller CPs derived from Leg2, named KTA and KTR, were effective in inactivating gram-positive bacteria such as *Staphylococcus aureus*. Their action mechanisms involve disrupting bacterial membranes and interfering with peptidoglycan synthesis [66]. More recently, Mahgoub et al. reported that trypsin-derived peptides from chickpea seed waste showed antibacterial activity against *Listeria innocua* and *Escherichia coli*, with inhibition zones of 22.45 and 25.35 mm, respectively [RS6].

#### Bioinformatic Analysis

Bioinformatics analyses were used in some studies with different objectives. In 11 studies, the analysis extended beyond enzyme inhibition assays to include molecular docking analyses, which are used to verify or predict the binding sites between CPs and the enzymes. Remarkably, Tan et al. [53] identified 71 peptides exhibiting a competitive or uncompetitive inhibition, while 6 peptides displayed a non-competitive type inhibition of α-amylase. Additionally, one study performed an in silico hydrolysate and molecular docking analysis to identify α-amylase inhibitory peptides from chickpea protein sequences [42]. This underscores the significance of bioinformatic tools for predicting the hydrolysis products and bioactivity of peptides and hydrolysates. In these cases, the validation of in silico predictions is essential to ensure that computational models accurately reflect experimental outcomes.

### Biological Impact of CPHs

#### In Vitro Studies: Cellular Models

Cellular Viability Evaluation

Cell viability assays were mainly used to assess the safety of hydrolysate exposure and, in cancer-related models, to evaluate potential antiproliferative effects. Among these approaches, the MTT (3-(4,5-dimethyl-2-thiazolyl)-2,5-diphenyl-2 H-tetrazolium bromide) assay was the most frequently used method to estimate metabolic activity as an indirect indicator of cell viability.

Using the MTT assay, various CPH fractions or CPs were found to be non-cytotoxic in several models, including murine macrophage RAW 264.7 cells [14, 67], human colon epithelial Caco-2 cells (at concentrations below 2000 µM for Leg1 peptide and 1000 µM for Leg2 peptide) [64], primary mouse spleen cells [68], and human monocyte THP-1 cell line [69, 70].

Another study aimed to evaluate CPHs as photoprotective ingredients. Using the Neutral Red Uptake assay, BJ fibroblast viability was at concentrations of 6.25–100 µg/mL after 48 h. Moreover, the 3.5–7 kDa peptide fraction from heat-treated chickpea hydrolysates exhibited the strongest fibroblast growth-stimulating activity [29].

On the other hand, MTT assay was used to evaluate the anticancer activity of CPs. So, oxovanadium-CPs inhibited cell growth in human lung cancer A549 cells [56]. Giron-Calle et al. [71] demonstrated that Pepsin–pancreatin CPHs inhibited the growth of Caco-2 cells by up to 45% and THP-1 cells by up to 78%, suggesting significant anticancer effects. Immunomodulatory activity of CPHs was showed in a co-culture of Caco-2 and THP-1 cells which a 66% increase in THP-1 proliferation was evidenced. Mahgoub et al. reported that trypsin-derived peptides from chickpea seed waste inhibited HepG2 and MCF-7 cancer cells by 83–86% at 100 µg/mL, with an IC₅₀ of 20 µg/mL [RS6].

An alternative approach commonly used for viability evaluation is the Sulforhodamine B (SRB) assay, valued for its cost-effectiveness and simplicity since it does not depend on metabolic activity measurements. Using this assay, Meza Márquez et al. [72] reported low cytotoxicity of ultrafiltrate of CPHs in murine peritoneal macrophages.

Additionally, the antiproliferative effects of CPHs were demonstrated by SRB, with accession BDN-9-3 showing the strongest activity (IC50 of 0.60 mg/mL in MCF-7, and of 0.63 mg/mL in MDA-MB-231 breast cancer cells), while other accessions ranged between a IC50 value of 0.69 to 1.15 mg/mL [62]. The SRB assay was also applied to human endometrial adenocarcinoma (Ishikawa) and breast cancer (MCF-7) cell lines. CPHs from *C. arietinum* and *C. reticulatum* showed dose-dependent cytotoxicity, with IC50 values of 0.36 and 0.29 mg/mL in Ishikawa cells, respectively, and 0.47 and 0.41 mg/mL in MCF-7 cells, respectively. Morphological analysis and DNA fragmentation further suggested that these hydrolysates may induce apoptosis in cancer cells [62].

### Antioxidant Potential of CPHs

The cellular antioxidant potential of CPHs and CPs was evaluated in five studies using cell-based approaches, mainly the cellular antioxidant activity (CAA) assay, together with viability or metabolic activity assays such as MTT. While MTT is not a direct antioxidant assay, it provides complementary information on cell viability and metabolic activity, helping to distinguish antioxidant effects from cytotoxic responses. In contrast, the CAA assay evaluates the ability of compounds to reduce intracellular oxidation by inhibiting the conversion of non-fluorescent dichlorodihydrofluorescein (DCFH) into fluorescent dichlorofluorescein (DCF), a reaction induced by peroxyl radicals generated from 2,2′-azobis(2-amidinopropane) dihydrochloride (ABAP).

In primary human dermal fibroblasts (HDFa), CPH fractions below 10 kDa reduced UVA-induced collagen degradation, suggesting a potential protective effect against photoaging-related oxidative damage. The CAA assay further supported the cellular antioxidant potential of these chickpea-derived fractions [16]. Similarly, in Caco-2 cells, CPs showing the strongest FRAP and DPPH activities in chemical assays also exhibited the highest antioxidant effects in the CAA assay, supporting the relevance of combining biochemical and cell-based antioxidant evaluations [41].

In addition to CAA-based approaches, one study evaluated the antioxidant potential of chickpea-derived peptides by measuring antioxidant enzyme activity and the regulation of genes involved in ROS metabolism. Guo et al. [73] reported that the chickpea-derived pentapeptide NRYHE increased the activity of catalase, glutathione peroxidase, and glutathione reductase, and stimulated the expression of Nrf2-regulated genes, including NQO1, HO-1, and γ-GCS, in Caco-2 and HT-29 cells exposed to H₂O₂-induced oxidative stress.

#### Anti-inflammatory and Immunomodulatory Activity

Eight studies evaluated the anti-inflammatory or immunomodulatory activity of CPHs and CPs using cell-based models, mainly macrophage-like or immune-related systems. These studies assessed NO production, pro-inflammatory cytokines, oxidative stress markers, and genes involved in inflammatory signaling. Several CPH fractions reduced NO production in activated macrophage models, including RAW 264.7 cells, where Xu et al. [67] reported inhibition after supplementation with 2 mg/mL hydrolysate. Bromelain-derived CPH also showed stronger NO inhibition than Alcalase-derived CPH, with reductions of 56% and 40%, respectively [14, 72].

Cytokine modulation was reported in four studies. Meza Márquez et al. [72] showed that fractions below 10 kDa reduced TNF-α and IL-1β at high concentrations, whereas fractions below 5 kDa inhibited IL-1β across all tested concentrations. Other studies reported that CPH effects may depend on co-exposure to bioactive compounds such as vitamin D [70], or on the inflammatory context. Rodríguez-Martín et al. [69] reported increased NO and ROS production together with reduced IL-1β, NF-κB, and NLRP3 expression, and enhanced SOD2 activity in a low-grade inflammation model. Overall, these findings suggest that CPHs and CPs may act as context-dependent immunomodulators rather than simple anti-inflammatory agents, with effects influenced by cell type, inflammatory stimulus, peptide size, dose, exposure time, and coexisting bioactive compounds.

### Antidiabetic Activity of CPHs

Several cellular biomarkers were used to evaluate the antidiabetic potential of CPHs and CPs. Glucose uptake was assessed in two studies, sucrase–maltase–isomaltase activity in one study, and DPP-IV inhibition in one study. Sucrase–maltase–isomaltase is a key intestinal brush-border enzyme complex involved in carbohydrate hydrolysis and glucose release, whereas DPP-IV is a serine protease that degrades incretin hormones and therefore contributes to the regulation of postprandial insulin secretion.

Chandrasekaran and Gonzalez de Mejia [11] reported that peptides such as VVFW, GEAGR, and FDLPAL modulated glucose-related responses in a dose-dependent manner. In silico-selected CPs, including AAWPGHPEF, IAIPPGIPYW, and PPGIPYW, also showed relevant DPP-IV inhibitory activity [52]. More recently, Oishi et al. [74] showed that REGDIIAVPTGVVF (CPP737) significantly enhanced glucose uptake in murine C2C12 myotubes in a dose-dependent manner, promoted AMPK phosphorylation, and induced GLUT4 translocation without affecting Akt phosphorylation. Together, CPHs may support glucose regulation by targeting digestive/incretin-related enzymes and promoting skeletal muscle glucose uptake.

#### Sensory-related Cell-based Assays: Bitter Taste Receptors

One study evaluated the effects of CPHs on extra-oral bitter receptor signaling in Caco-2 cells. CPH exposure reduced T2R4 and increased T2R14 expression, while downstream signaling markers were generally reduced at higher concentrations [11]. These receptor-specific effects do not establish sensory bitterness or acceptability, which require direct sensory validation. In this sense, Garza-Aguilar et al. [RS9] found that intensive Flavourzyme treatment of germinated, Se-biofortified chickpea (4% E/S, 6 h) increased umami-associated amino acids while retaining sweet amino acids that may mask bitterness, although bitterness was not directly assessed.

### In Vivo Studies: Animal Models

#### Anticancer Potential of CPHs

Two studies provided evidence supporting the anticancer potential of CPHs in animal models. Sánchez-Chino et al. [48] reported that CPH supplementation exerted a protective effect against colon carcinogenesis in an azoxymethane (AOM)-induced mouse model, whereas Xue et al. [68] described protective effects against hepatic carcinoma in H-22 tumor-bearing KM mice. In both studies, CPH administration was associated with favourable changes in tumor-related outcomes, supporting the potential role of chickpea-derived hydrolysates as bioactive dietary components in experimental cancer models.

### Antihypertensive Potential of CPHs

The antihypertensive potential of CPHs was evaluated in three studies using male spontaneously hypertensive rats (SHRs). Chávez-Ontiveros et al. [33] reported that CPHs derived from extruded chickpea flour reduced systolic blood pressure, while an optimized Alcalase-derived CPH also exerted significant antihypertensive effects in vivo [60]. These findings are consistent with previous in vitro ACE-inhibitory assays using chickpea-derived hydrolysates or peptides [12, 22, 30, 46, 59], as well as with in silico analyses predicting more than 300 ACE-inhibitory peptides derived from chickpea legumin and provicilin sequences [42]. More recently, five-week administration of an optimized Alcalase-derived CPH (50 mg/kg bw/day) to SHR reduced systolic, diastolic, and mean blood pressure, with a maximum systolic reduction of 39.80 mmHg, and increased renal ACE2 and Mas1 expression [RS5].

### Antioxidant and Anti-Inflammatory Activity of CPHs

The anti-inflammatory potential of Alcalase-derived CPH was evaluated in a BALB/c mouse model of croton oil-induced ear edema. In this model, topical administration of 10 µg/ear of CPH reduced ear edema, reaching 62% inhibition [24]. Radlowski et al. [61] further reported that in vivo administration of CPH to high-fat diet-fed C57BL/6J mice decreased the non-alcoholic fatty liver disease activity score and reduced inflammatory cell infiltration. In the same study, plasma adiponectin levels increased, whereas inflammatory markers such as CXCL1 and CCL25 were downregulated, supporting the potential anti-inflammatory effects of CPH in diet-induced metabolic dysfunction.

## Antidiabetic Activity of CPHs

The antidiabetic potential of CPHs has been explored mainly through biochemical, cell-based, and ex vivo approaches, while fewer studies have assessed these effects in animal models. In BALB/c mice, Navarro-Leyva et al. [24] reported that pepsin–pancreatin-hydrolyzed chickpea proteins preserved antihyperglycemic activity at 10 mg/kg, whereas Alcalase-derived CPH was more effective in reducing glucose levels. Radlowski et al. [61] also showed that CPH administration to high-fat diet-fed C57BL/6J mice exerted insulin-sensitizing effects, associated with the regulation of signaling-related genes such as *Pik3r1*, *Ikbkb*, and *Map2k1*. In a type 2 diabetes-like rat model, oral administration of chickpea albumin hydrolysate (200 or 400 mg/kg bw/day for 30 days) improved glycemic control, lipid profile, antioxidant status, pancreatic histology, and hepatic glucose-metabolism markers [RS7]. These results suggest that CPHs can influence glucose homeostasis through both direct antihyperglycemic effects and modulation of insulin-related pathways.

### Lipid Metabolism and Metabolic Health Effects of CPHs

Several animal studies included in the systematic review evaluated the effects of CPHs on lipid metabolism. Animals fed a high-fat diet supplemented with CPHs showed reduced LDL-cholesterol and triglyceride levels, with limited changes in HDL-cholesterol, suggesting a potential hypolipidemic effect [48, 68]. More recently, Radlowski et al. [61] demonstrated that CPH administration to high-fat diet-fed C57BL/6J mice reduced body weight gain, adipocyte hypertrophy, and hepatic steatosis in a dose-dependent manner at 400–800 mg/kg body weight. This treatment was also associated with the downregulation of lipogenesis-related genes, including *Acaca*, which encodes acetyl-CoA carboxylase alpha, a key enzyme involved in fatty acid synthesis.

As complementary evidence outside the systematic review, Yahia et al. [75] reported that CPHs with a degree of hydrolysis of 17% reduced serum cholesterol, decreased both LDL- and HDL-cholesterol, and increased APOA4 concentrations in cholesterol-fed rats. Although this study was not included in the systematic review, its findings support the potential relevance of CPHs in cholesterol metabolism.

### Human Evidence and Clinical Translation Gap

To date, no human intervention studies have directly evaluated the effects of CPHs or purified CPs. Human studies with whole chickpeas or legume-based dietary patterns provide only contextual evidence, suggesting potential metabolic benefits that cannot be directly attributed to chickpea-derived peptides [76, 77]. Similarly, clinical studies with protein hydrolysates from other plant sources support the broader translational interest of plant-derived bioactive peptides, but these findings cannot be extrapolated to CPHs without specific validation [78].

Although findings from in vitro and animal models cannot be directly extrapolated to establish dosing regimens or methodological conditions for human clinical trials, they may provide a useful starting point for their design. In particular, the dose ranges evaluated in animal studies, together with evidence obtained from simulated gastrointestinal digestion, may help guide the selection of initial doses and identify potential bioaccessibility bottlenecks. Future human studies should also consider the influence of the food matrix on peptide release, stability, and absorption, as well as technological strategies such as microencapsulation to mask bitterness and improve the sensory acceptability of chickpea-derived bioactive ingredients.

## Functional and Health Implications

Taken together, the available evidence positions CPHs and CPs as multifunctional food-grade ingredients of interest for functional foods, nutritional supplements, and specialized dietary formulations. Their relevance extends beyond classical health-oriented bioactivities, as emerging evidence suggests possible roles in alcohol metabolism through ADH modulation and in food preservation through antifungal and antibacterial peptides. These applications remain preliminary but support the broader interest of CPHs in plant protein valorization, food functionality, and clean-label innovation.

## Limitations of the Literature and Research Gaps

Despite the relevant evidence summarized in this review, the field remains limited by heterogeneous terminology, variable hydrolysis protocols, inconsistent reporting of degree of hydrolysis and bioactivity outcomes, and incomplete peptide-level characterization. Most studies are still based on biochemical assays, simulated digestion models, cell cultures, or animal models, while human intervention trials, sensory validation, regulatory assessment, and industrial scale-up remain scarce. These limitations should be considered when interpreting the current evidence and define the main priorities for future research.

## Conclusion

This review highlights the potential of CPHs and CPs as candidate functional plant-based ingredients. The integration of network analysis and systematic review showed that current research is mainly focused on antioxidant, antihypertensive, antidiabetic, anti-inflammatory, lipid-lowering, immunomodulatory, antimicrobial, and anticancer-related activities. These effects are supported primarily by biochemical assays, simulated digestion models, cell cultures, and animal studies, indicating that the field remains largely preclinical.

Future research should prioritize standardized terminology and protocols, diversification of proteolytic enzymes, peptide-level characterization, bioavailability assessment, validation in real food matrices, sensory evaluation, scale-up studies, and well-designed human trials to determine whether the available preclinical evidence can be translated into practical nutritional and health-related applications.

## Supplementary Information

Below is the link to the electronic supplementary material.

Supplementary Material 1 Graphical abstract. The scheme summarizes the main experimental stages reported in the literature, from the selection of chickpea raw materials and protein fractions to alternative pre-treatments, protein isolation and extraction, enzymatic hydrolysis, downstream refining, and product characterization. Proteolysis approaches include microbial, digestive, and plant-derived proteases, under variable hydrolysis conditions. Subsequent processing may involve enzyme inactivation, molecular-weight fractionation, ultrafiltration, chromatography, and final stabilization. Characterization strategies include assessment of the degree of hydrolysis, amino acid composition, mass spectrometry, in silico analyses, and biochemical, cellular, animal, and static simulated gastrointestinal digestion (SGID) models. The main sources of methodological variability and critical processing factors identified across studies are summarized in the right-hand panel

High Resolution Image (TIFF 685KB)

Supplementary Material 2 Integrated biological activities of chickpea protein hydrolysates and derived peptides across levels of experimental evidence. The figure summarizes the main reported cardiometabolic, antioxidant, anti-inflammatory, antiproliferative, immunomodulatory, antimicrobial, and metal-binding activities of chickpea protein hydrolysates (CPHs) and derived peptides. Reported mechanisms include modulation of blood pressure, glucose and lipid metabolism, radical scavenging, metal chelation, antioxidant defenses, and inflammatory pathways. The evidence ladder indicates the level of experimental support, from in silico predictions and biochemical assays to cell and animal models; no human intervention studies were identified

High Resolution Image (TIFF 510KB)

Online resource 1

## Data Availability

The data supporting this review are derived from previously published studies cited in the manuscript. The bibliographic records retrieved from PubMed and Scopus, the processed dataset used for the network analysis, and the thesaurus file used for term harmonization are available from the corresponding author upon reasonable request. Redistribution of raw Scopus records may be subject to database licensing restrictions.

## Abbreviations

ABAP: 2,2′-azobis(2-amidinopropane) dihydrochloride
ABTS: 2,2′-azinobis(3-ethylbenzothiazoline-6-sulfonic acid)
ACE: angiotensin-converting enzyme
ACE-I: angiotensin-converting enzyme inhibitory activity
ADH: alcohol dehydrogenase
ADMET: absorption, distribution, metabolism, excretion, and toxicity
Akt: protein kinase B
ALP: alkaline phosphatase
AMPK: AMP-activated protein kinase
AOM: azoxymethane
APOA4: apolipoprotein A-IV
BHT: butylated hydroxytoluene
CAA: cellular antioxidant activity
CCL25: C-C motif chemokine ligand 25
CPHs: chickpea protein hydrolysates
CPI: chickpea protein isolate
CPs: chickpea-derived peptides
CXCL1: C-X-C motif chemokine ligand 1
DCF: dichlorofluorescein
DCFH: dichlorodihydrofluorescein
DH: degree of hydrolysis
DPPH: 2,2-diphenyl-1-picrylhydrazyl
DPP-IV: dipeptidyl peptidase IV
DRV: dietary reference value
E/S: enzyme/substrate ratio
FRAP: ferric reducing antioxidant power
GLUT2: glucose transporter 2
GLUT4: glucose transporter 4
GSH: reduced glutathione
HDFa: human dermal fibroblasts
HDL: high-density lipoprotein
HHP: high hydrostatic pressure
HMG-CoA: 3-hydroxy-3-methylglutaryl coenzyme A
HO-1: heme oxygenase 1
HYA: hyaluronidase
IC50: half-maximal inhibitory concentration
IL-1β: interleukin-1 beta
Inact.: inactivation
INFOGEST: international network of excellence on the fate of food in the gastrointestinal tract
LAPU: leucine aminopeptidase units
LDL: low-density lipoprotein
LOX: lipoxygenase
MAPK: mitogen-activated protein kinase
MDA: malondialdehyde
MTT: 3-(4,5-dimethyl-2-thiazolyl)-2,5-diphenyl-2 H-tetrazolium bromide
NAFLD: non-alcoholic fatty liver disease
NF-κB: nuclear factor kappa B
NLRP3: NOD-,LRR-,and pyrin domain-containing protein 3
NO: nitric oxide
NQO1: NAD(P)H quinone dehydrogenase 1
Nrf2: nuclear factor erythroid 2-related factor 2
ORAC: oxygen radical absorbance capacity
O/W: oil-in-water
PDCAAS: protein digestibility-corrected amino acid score
PRISMA: Preferred Reporting Items for Systematic Reviews and Meta-Analyses
RH: relative humidity
ROS: reactive oxygen species
SGID: simulated gastrointestinal digestion
SHRs: spontaneously hypertensive rats
SOD: superoxide dismutase
SOD2: superoxide dismutase 2
SRB: sulforhodamine B
TBARS: thiobarbituric acid reactive substances
T2R4: taste receptor type 2 member 4
T2R14: taste receptor type 2 member 14
TNF-α: tumor necrosis factor alpha
UVA: ultraviolet A
w/o: water-in-oil
w/v: weight/volume
w/w: weight/weight
γ-GCS: gamma-glutamylcysteine synthetase
