# Supplementary material for: Functional and Nutritional Potential of Chickpea Protein Hydrolysates: A Systematic Review and Plant-protein Network Analysis
Source: Plant Foods Hum Nutr. 2026 Sep 2;81(3):119. doi: 10.1007/s11130-026-01568-z (PMC13558302; doi:10.1007/s11130-026-01568-z)
Supplement: Supplementary file 1 [file 11130_2026_1568_MOESM3_ESM.docx]

**Online Resource 1**

Supplementary material for:

**Functional and Nutritional Potential of Chickpea Protein Hydrolysates: A Systematic Review and Plant-Protein Network Analysis**

Noelia M. Rodríguez-Martín ^1,2*^ [nmrodriguez@uloyola.es](mailto:nmrodriguez@uloyola.es)

José Carlos Márquez-López ^2^ [jcmarquez@ig.csic.es](mailto:jcmarquez@ig.csic.es)

María-Soledad Fernández-Pachón ^3^ [msferpac@upo.es](mailto:msferpac@upo.es)

Justo Pedroche ^2*^ [j.pedroche@csic.es](mailto:%20j.pedroche@csic.es)

^1^ Department of Biological and Health Sciences, Faculty of Health Sciences, Loyola University (Universidad Loyola Andalucía), Avda. de las Universidades s/n, 41704 Dos Hermanas, Spain

^2^ Instituto de la Grasa-CSIC, Plant Protein Group, Avda. Rectora Rosario Valpuesta nº 1, 41089 Dos HermanasSeville, Spain.

^3^ Área de Nutrición y Bromatología, Departamento de Biología Molecular e Ingeniería Bioquímica, Universidad Pablo de Olavide, Avda. Rectora Rosario Valpuesta nº 1, 41089 Dos Hermanas Seville, Spain.

^*^Corresponding author NMRM and JP.

**Online Resource 1**

Additional methodological details and extended tables supporting the systematic review and network analysis of chickpea protein hydrolysates. This file includes the cluster terms identified in the network analysis, PRISMA checklist, detailed summaries of *in vitro*, cell-based, and animal studies on chickpea protein hydrolysates, and complementary tables on chickpea seed composition, including macronutrients, micronutrients, lipid profile, and amino acid profile.

**List of supplementary material, figures and tables**

[M1. Terms included in the final title–abstract co-occurrence network, grouped according to the VOSviewer cluster assignment	3](#_Toc239090351)

[Fig S1. Overview of chickpea protein hydrolysate production, characterization, and major sources of methodological variability.	4](#_Toc239090352)

[Fig S2. Integrated biological activities of chickpea protein hydrolysates and derived peptides across levels of experimental evidence.	4](#_Toc239090353)

[Table S1. Adherence to the PRISMA 2020 checklist for systematic reviews.	5](#_Toc239090354)

[Table S2. Overview of *in vitro* studies on chickpea protein hydrolysates (CPHs), including raw materials, pre-treatments, hydrolysis conditions, and key findings about bioactivity.	10](#_Toc239090355)

[Table S3. Description of cellular and *in vivo* studies on chickpea protein hydrolysates (CPHs): raw materials, pre-treatments, hydrolysis conditions, cellular line or animal model, and key findings.	21](#_Toc239090356)

[Table S4. Proximal Macronutrient Composition of Raw Chickpeas.	30](#_Toc239090357)

[Table S5. Vitamin and Mineral Content of Raw Chickpeas.	31](#_Toc239090358)

[Table S6. Lipid Profile of Raw Chickpeas.	32](#_Toc239090359)

[Table S7. Amino acid profile of Raw Chickpea Flour.	33](#_Toc239090360)

[Supplementary References	34](#_Toc239090361)

[Supplementary References included in the manuscript as RS	34](#_Toc239090362)

# M1. Terms included in the final title–abstract co-occurrence network, grouped according to the VOSviewer cluster assignment

Terms were extracted from titles and available abstracts using binary counting. Synonyms and spelling variants were merged using a thesaurus, while general vocabulary, measurement units, non-plant protein terms, and unrelated topics were excluded. The final manually curated network comprised 96 connected terms distributed across six clusters.

**Cluster 1 (26 terms)**: antioxidant, seed, in vitro, bioactive peptides, ACE inhibition, rat model, in silico, in vitro digestion, in vivo, oxidative stress, hemp, bioavailability, quinoa, antihypertensive activity, metal-chelating activity, hypertensive rat model, chia, animal model, thermolysin, antidiabetic activity, DPP-IV inhibition, alpha-glucosidase inhibition, alpha-amylase inhibition, savinase, actinidin, and teff.

**Cluster 2 (23 terms)**: soy, degree of hydrolysis, protein solubility, techno-functional properties, emulsifying properties, particle size, surface hydrophobicity, physicochemical properties, foaming capacity, color, zeta potential, viscosity, emulsion stability, bitterness, sensory properties, thermal stability, secondary structure, rheology, foam stability, water-holding capacity, gelation, oil-holding capacity, and sesame.

**Cluster 3 (20 terms)**: cell model, wheat, rice, human study, maize, cereal, mouse model, reactive oxygen species, cytotoxicity, Caco-2, macrophages, anti-inflammatory activity, barley, HepG2, RAW 264.7, anticancer activity, immunomodulatory activity, clinical trial, randomized controlled trial, and pronase.

**Cluster 4 (11 terms)**: bean, amino acid composition, protein digestibility, pea, legume, chickpea, mung bean, peanut, lentil, lupin, and faba bean.

**Cluster 5 (11 terms)**: protease, Alcalase, Flavourzyme, papain, Neutrase, bromelain, Protamex, peptide profile, sunflower, Corolase, and ficin.

**Cluster 6 (5 terms)**: pepsin, trypsin, pancreatin, antimicrobial activity, and chymotrypsin.

# Fig S1. Overview of chickpea protein hydrolysate production, characterization, and major sources of methodological variability.

The scheme summarizes the main experimental stages reported in the literature, from the selection of chickpea raw materials and protein fractions to alternative pre-treatments, protein isolation and extraction, enzymatic hydrolysis, downstream refining, and product characterization. Proteolysis approaches include microbial, digestive, and plant-derived proteases, under variable hydrolysis conditions. Subsequent processing may involve enzyme inactivation, molecular-weight fractionation, ultrafiltration, chromatography, and final stabilization. Characterization strategies include assessment of the degree of hydrolysis, amino acid composition, mass spectrometry, in silico analyses, and biochemical, cellular, animal, and static simulated gastrointestinal digestion (SGID) models. The main sources of methodological variability and critical processing factors identified across studies are summarized in the right-hand panel.

# Fig S2. Integrated biological activities of chickpea protein hydrolysates and derived peptides across levels of experimental evidence.

# Table S1. Adherence to the PRISMA 2020 checklist for systematic reviews.

| **Section and Topic** | **Item #** | **Checklist item** | **Location where item is reported** |
| --- | --- | --- | --- |
| **TITLE** | | |  |
| Title | 1 | Identify the report as a systematic review. | Title page |
| **ABSTRACT** | | |  |
| Abstract | 2 | See the PRISMA 2020 for Abstracts checklist. | Abstract |
| **INTRODUCTION** | | |  |
| Rationale | 3 | Describe the rationale for the review in the context of existing knowledge. | Introduction |
| Objectives | 4 | Provide an explicit statement of the objective(s) or question(s) the review addresses. | End of introduction |
| **METHODS** | | |  |
| Eligibility criteria | 5 | Specify the inclusion and exclusion criteria for the review and how studies were grouped for the syntheses. | Methods, Systematic Review |
| Information sources | 6 | Specify all databases, registers, websites, organisations, reference lists and other sources searched or consulted to identify studies. Specify the date when each source was last searched or consulted. | Methods, Network Analysis and Systematic Review |
| Search strategy | 7 | Present the full search strategies for all databases, registers and websites, including any filters and limits used. | Methods, Network Analysis and Systematic Review |
| Selection process | 8 | Specify the methods used to decide whether a study met the inclusion criteria of the review, including how many reviewers screened each record and each report retrieved, whether they worked independently, and if applicable, details of automation tools used in the process. | Methods, Systematic Review; Fig. 1 |
| Data collection process | 9 | Specify the methods used to collect data from reports, including how many reviewers collected data from each report, whether they worked independently, any processes for obtaining or confirming data from study investigators, and if applicable, details of automation tools used in the process. | Methods, Systematic Review; Online Resource 1, Tables S2–S3 |
| Data items | 10a | List and define all outcomes for which data were sought. Specify whether all results that were compatible with each outcome domain in each study were sought (e.g. for all measures, time points, analyses), and if not, the methods used to decide which results to collect. | Methods, Systematic Review; Results and Discussion; Online Resource 1, Tables S2–S3 |
|  | 10b | List and define all other variables for which data were sought (e.g. participant and intervention characteristics, funding sources). Describe any assumptions made about any missing or unclear information. | Methods, Systematic Review; Online Resource 1, Tables S2–S3 |
| Study risk of bias assessment | 11 | Specify the methods used to assess risk of bias in the included studies, including details of the tool(s) used, how many reviewers assessed each study and whether they worked independently, and if applicable, details of automation tools used in the process. | Not performed; the review included highly heterogeneous biochemical, in silico, cell-based, and animal studies for which no single risk-of-bias tool was applicable |
| Effect measures | 12 | Specify for each outcome the effect measure(s) (e.g. risk ratio, mean difference) used in the synthesis or presentation of results. | Not applicable; no meta-analysis was performed. Outcomes were summarized as reported in the original studies |
| Synthesis methods | 13a | Describe the processes used to decide which studies were eligible for each synthesis (e.g. tabulating the study intervention characteristics and comparing against the planned groups for each synthesis (item #5)). | Methods, Systematic Review; studies were grouped by methodological approach and bioactivity area |
|  | 13b | Describe any methods required to prepare the data for presentation or synthesis, such as handling of missing summary statistics, or data conversions. | Online Resource 1, Tables S2–S3; no data transformation for meta-analysis was performed |
|  | 13c | Describe any methods used to tabulate or visually display results of individual studies and syntheses. | Fig. 1; Fig. 2; Online Resource 1, Tables S2–S3 |
|  | 13d | Describe any methods used to synthesize results and provide a rationale for the choice(s). If meta-analysis was performed, describe the model(s), method(s) to identify the presence and extent of statistical heterogeneity, and software package(s) used. | Methods, Systematic Review; narrative synthesis was performed because of methodological heterogeneity |
|  | 13e | Describe any methods used to explore possible causes of heterogeneity among study results (e.g. subgroup analysis, meta-regression). | Not applicable |
|  | 13f | Describe any sensitivity analyses conducted to assess robustness of the synthesized results. | Not applicable |
| Reporting bias assessment | 14 | Describe any methods used to assess risk of bias due to missing results in a synthesis (arising from reporting biases). | Not applicable |
| Certainty assessment | 15 | Describe any methods used to assess certainty (or confidence) in the body of evidence for an outcome. | Not formally assessed; evidence strength was discussed qualitatively according to model type and translational relevance |
| **RESULTS** | | |  |
| Study selection | 16a | Describe the results of the search and selection process, from the number of records identified in the search to the number of studies included in the review, ideally using a flow diagram. | Fig. 1; Methods, Systematic Review |
|  | 16b | Cite studies that might appear to meet the inclusion criteria, but which were excluded, and explain why they were excluded. | Fig. 1; full-text exclusions summarized by reason |
| Study characteristics | 17 | Cite each included study and present its characteristics. | Online Resource 1, Tables S2–S3 |
| Risk of bias in studies | 18 | Present assessments of risk of bias for each included study. | Not performed; see Item 11 |
| Results of individual studies | 19 | For all outcomes, present, for each study: (a) summary statistics for each group (where appropriate) and (b) an effect estimate and its precision (e.g. confidence/credible interval), ideally using structured tables or plots. | Results and Discussion; Online Resource 1, Tables S2–S3 |
| Results of syntheses | 20a | For each synthesis, briefly summarise the characteristics and risk of bias among contributing studies. | Results and Discussion; Online Resource 1, Tables S2–S3. Risk of bias was not formally assessed |
|  | 20b | Present results of all statistical syntheses conducted. If meta-analysis was done, present for each the summary estimate and its precision (e.g. confidence/credible interval) and measures of statistical heterogeneity. If comparing groups, describe the direction of the effect. | Not applicable |
|  | 20c | Present results of all investigations of possible causes of heterogeneity among study results. | Not applicable |
|  | 20d | Present results of all sensitivity analyses conducted to assess the robustness of the synthesized results. | Not applicable |
| Reporting biases | 21 | Present assessments of risk of bias due to missing results (arising from reporting biases) for each synthesis assessed. | Not applicable |
| Certainty of evidence | 22 | Present assessments of certainty (or confidence) in the body of evidence for each outcome assessed. | Not formally assessed; limitations and translational evidence level are discussed in Results and Discussion, Limitations, and Conclusion |
| **DISCUSSION** | | |  |
| Discussion | 23a | Provide a general interpretation of the results in the context of other evidence. | Results and Discussion; Conclusion |
|  | 23b | Discuss any limitations of the evidence included in the review. | Limitations of the Literature and Research Gaps |
|  | 23c | Discuss any limitations of the review processes used. | Limitations of the Literature and Research Gaps |
|  | 23d | Discuss implications of the results for practice, policy, and future research. | Functional and Health Implications; Limitations; Conclusion |
| **OTHER INFORMATION** | | |  |
| Registration and protocol | 24a | Provide registration information for the review, including register name and registration number, or state that the review was not registered. | Not registered |
|  | 24b | Indicate where the review protocol can be accessed, or state that a protocol was not prepared. | No protocol was prepared |
|  | 24c | Describe and explain any amendments to information provided at registration or in the protocol. | Not applicable |
| Support | 25 | Describe sources of financial or non-financial support for the review, and the role of the funders or sponsors in the review. | Declarations and statements |
| Competing interests | 26 | Declare any competing interests of review authors. | Declarations and statements |
| Availability of data, code and other materials | 27 | Report which of the following are publicly available and where they can be found: template data collection forms; data extracted from included studies; data used for all analyses; analytic code; any other materials used in the review. | Declarations and statements |

Checklist adapted from Page et al. [S1].

# Table S2. Overview of *in vitro* studies on chickpea protein hydrolysates (CPHs), including raw materials, pre-treatments, hydrolysis conditions, and key findings about bioactivity.

| **Raw Material** | **Pre-treatments, defatting and precipitation methods** | **Hydrolysis conditions and other treatments (digestions or incubations)** | **Main Results** | **Source** |
| --- | --- | --- | --- | --- |
| Chickpea seeds (cv Athenas) | Alkaline solubilization (pH 12) and protein precipitation at isoelectric point (pH 4.3). | Sequential hydrolysis:  -Alcalase 2.4 L (E/S 0.3 AU/g protein, 60 min, pH 7.0).  -Flavourzyme 1000 MG (E/S 50 LAPU/g protein, 90 min, pH 8.0). Inact.: acid pH. | Peptides with strong ACE inhibitory activity (IC50 0.105–0.117 mg/mL), suggesting potential as functional antihypertensive ingredients.  Four main fractions were collected. Fraction 2: 50.5 to 58 mL showed the highest inhibitory activity; contained peptides of 800 to 1000 Da approx. 6 to 8 amino acids. | [35] |
| Chickpea seeds (cv Athenas) | Defatting (hexane).  Protein extraction with borate buffer (0.1 M, pH 8.3) to isolate the globulin fraction.  Purification by ion-exchange chromatography and gel filtration to obtain legumin. | Alcalase (2.4 L, 0.3 AU/g), 50 °C, pH 7, 30 min.  Inact.: acidification to pH 5. | Chickpea hydrolysates exhibited strong ACE inhibitory activity (IC50 0.18 mg/mL), while purified peptides showed higher potency (IC50 0.011–0.021 mg/mL)  ACE inhibitory sequences with the amino acids:  M, D: IC50 0.021 mg/mL; M, D, P, F, L, I: IC50 0.011 mg/mL; M (this peak does not represent a free amino acid): IC50 0.020 mg/mL; M, F, D, L: IC50 0.013 mg/mL; M, D, L: IC50 0.021 mg/mL; M, D, L, A: IC50 0.013 mg/mL. | [36] |
| Chickpea seeds | Alkaline solubilization (pH 12) and protein precipitation at isoelectric point (pH 4.3). | Sequential hydrolysis of CPI 5 % (w/v):  -Alcalase 2.4 L (E/S: 0.3 AU/g), 50 °C, pH 8, 60 min.  -Flavourzyme (E/S: 100 LAPU/g), 50 °C, pH 7, 200 min.  Inact.: 80 °C for 20 min.  Final filtration 0.45 µm and freeze-dried. | CPH showed high copper-chelating capacity and β-carotene oxidation inhibition related (but not caused) by histidine content. | [55] |
| Chickpea seeds | Defatted (hexane).  Solubilization (pH 10.5 in 0.25 % Na_2_SO_3_ (w/v)) and protein precipitation at isoelectric point (pH 4.3).  Spray-dried. | Sequential hydrolysis:  -Alcalase (4.6 mL /L, 50 °C, pH 8 for 1 h).  -Flavourzyme (1.85 mL/L, 50 °C, pH 7 for 2 h).  Inact.: 85 ºC, 10 min. | Significant hypocholesterolemic effect (*in vitro* micellar solubility of cholesterol) in the presence of CPHs, and antioxidant activity (β-carotene, FRAP, DPPH). | [9] |
| Kabuli (CDC Xena) and Desi (Myles) chickpea, and yellow pea (CDC Golden) | In pilot plant: Alkaline extraction (pH 9) and protein precipitation at isoelectric point (pH 4.5).  Defatted with hexane (only for chickpea varieties). | CPI 5 % (w/w).  Three hydrolysis conditions:  (1) Alcalase and Flavourzyme (AF), buffer Phosphate 0.01 M, pH 7.4, 50 °C, pH 7/8, E/S (w/w) 1/8/1/10, duration 1/1.5 h.  (2) Papain (P), buffer Phosphate 0.01 M, pH 6.8, 40 °C, pH 6.5, E/S (w/w) 1/25, duration 4 h.  (3) Gastrointestinal (GIS), buffer Glycine 0.01 M, pH 2, 37 °C, pH 2/6.5, E/S (w/w) 1/250, duration 2/2.5 h. | The ACE inhibitory activity (IC50) of CPH was: AF 316, GIS 229, P 282 µg/mL for kabuli varieties, and AF 228, GIS 140, P 180 µg/mL for desi varieties.  The GIS digestion values indicate that ACE inhibitory activity is retained after exposure to digestive enzymes.  Peptide blend; 50 percent of peptides in the less than 4 kDa range. | [45] |
| Dry chickpeas | Defatted with acetone powder (to remove phenolics and lipids compounds).  Extract of albumin and globulin fractions: solubilization at pH 9.5.  Preheated (85 °C, 30 min). | Pepsin hydrolysis (34 mg/g acetone powder), pH 2.5, 37 °C, 24 h.  Inact.: 85 °C, 30 min. | Fractions <10 kDa showed peptides showed high free radical scavenging activity Fe^+2^ (122-156 umol Na2EDTA/g powder) and high antioxidant activity (ABTS, 143-277 µmol trolox/g).  These fractions also showed lower iron chelation and foam/emulsion stability. | [20] |
| Chickpea seeds | Defatted with hexane.  Solubilization (pH 8) and protein precipitation at isoelectric point (pH 5), and neutralization at pH 7.  Spray dried.  HHP (100-600 MPa, 2–40 min, 50 °C). | During or after HHP treatment, Alcalase 2.4 L 1:20 (w/v) E/S ratio, 50 °C, pH 8, 360 min.  Inact.: 95 °C, 10 min. | High FRAP values and superoxide radical scavenging activity were obtained under HHP-assisted hydrolysis.  Optimal HHP conditions were 20 min at 200 MPa. | [32] |
| *Cicer Arietinum* L. variety Blanco Sinaloa 92  (Fresh and hard-to-cook seeds) | Cooked (140 min for fresh grains; 210 min for whole grains).  Protein precipitation at isoelectric point.  Globulin and albumin extraction (5 % K_2_SO_4_, pH 7, 25 °C, 60 min and subsequent dialyzed <10,000 cut off). | Three enzymatic conditions:  (1) Alcalase: 50 °C, pH 9.0, 2 h,  (2) Papain: 40 °C, pH 6.5, 2 h,  (3) Pancreatin: 39 °C, pH 8.0, 2 h.  After pH was adjusted to 4.0 and inactivated at 95 °C for 10 min. | High ACE-inhibitory and antioxidant (DPPH, ABTS) activities were obtained using both fresh and hard-to-cook chickpeas. | [30] |
| Chickpea | Defatting with ether,  Solubilization with distilled water, precipitation at pH 4.3, discard the precipitate, new precipitation at pH 3.7 for albumin extraction. | Subsequential hydrolysis:  -Alcalase at E/S ratio of 0.3 AU/g, 50 °C, pH 8.0, 1 h.  -Flavourzyme at E/S ratio of 50 LAPU/g, 50 °C, pH 7.0, 2 h.  Inact.: 80 °C for 10 min. (Xue et al 2012) | The low-molecular-weight fraction (Fra.III) was the most potent in all assays: DPPH inhibition 64.9 % at 5 mg/mL; hydroxyl radical inhibition 95.8 % at 5 mg/mL; TEAC (ABTS) 1.918 mmol/L at 5 mg/mL; FRAP A700: 0.058 at 5 mg/mL. Peptide identification: RQSHFANAQP (MW 1155 Da).  The increase in bitter value with higher degree of hydrolysis may represent a limitation. | [S2] |
| Chickpea, lupin, pea, lentils, bean, soybean seeds | Protein extraction in 100 mM Tris‑HCl / 0.5 M NaCl, pH 8.2, 2 h, 4 C.  Dialysis (24 h, 4 C). | Pepsin E/S ratio 1:100, 37 °C, pH 2, 18 h. | CPH showed ACE inhibitory activity with IC50 (673 ± 15 µg/mL). This was comparable to hydrolysates from common bean, lentil, and pea, while lupin and soybean exhibited lower values. | [59] |
| Chickpea flour | Defatting with hexane.  Cooking 80 °C for 5 min.  Protein solubilization (pH 9) and precipitation at isoelectric point (pH 4). | Alcalase (1:1 (U/mg) E/S) at 50 °C, pH 8, 210 min.  Inact.: 80 °C for 20 min. | Frac.III showed strong antioxidant activity: DPPH 67.32% at 200 µg/mL; β-carotene oxidation inhibition 66.92%; Fe²⁺ chelating activity 50.45% at 5 mg/mL.  Subfractions fractions of Fra.III: P3 and P8. Peptide identification: DHG, VGDI | [23] |
| Chickpea seeds | Solubilization (pH 8) and protein precipitation at pH 3.5.  Heated to 80 °C for 30 min. | Alcalase 1% E/S (v/v), 50 °C, pH 7, 10-210 min.  Inact.: 90 °C for 10 min.  O/W emulsions (50/50).  Post-hydrolysis SGID (Pepsin-Corolase method, by Walsh et al., 2004). | Emulsions of CPH showed increased antioxidant activity, with a moderate rise in FRAP and a much greater increase in ORAC. Emulsions after SGID also displayed high antioxidant activities.  ACE inhibitory activity decreased in the emulsion, whereas DPP-IV inhibition increased; both activities were improved after SGID treatment. | [22] |
| Kabuli chickpeas | Protein solubilization (pH 11.5), and precipitation at isoelectric point. | CPI 1:10 (w/v), two conditions:  (1) SGID: (Pepsin-pH 2- and pancreatin-pH 7.5, 1:50 E/S ratio) (*Torres-Fuentes et al., 2011*).  (2) Bromelain (1:50 E/S) pH 5.5 – 6.5, 55 °C for 30 min.  Inact.: boiling 1 min. | SGID hydrolysates (20 peptides): PHPATSGGGL, YVDGSGTPLT, SPQSPPFATPLW; Bromelain hydrolysates (12 peptides): KMTAGSGVT, GKAAPGSGGGTKA, GLTQGASLAGSGAPSPLF.  Peptides showed in silico antidiabetic potential, and hydrolysates demonstrated DPP-IV (IC 50) (SGID: 245 ug/mL, Bromelain: 790 ug/mL, both at 10 mg/mL), and α-amylase inhibitory activities (SGID: 38.4±1.4 % SGID, Bromelain: 11.0±0.8 %, both at 10 mg/mL).  Bitterness sequences were in silico detected. | [44] |
| Legume proteins (including chickpeas) | Autoclaved for 20 min at 121 °C. | Two hydrolysis conditions:  (1) SGID with Pepsin/substrate (1:20 (w/w)), pH 2; and pancreatin/substrate (1:20 (w/w)), pH 7.5, 2 h.  Inact.: 75 °C, 20 min  (2) Alcalase hydrolysis 1:20 (w/w). 50 °C, pH 7, 2h.  Inact.: 75 °C, 20 min. | CPHs from Alcalase showed the strongest NO radical scavenging activity and high ABTS values, while SGID hydrolysates exhibited higher DPPH activity and high HMG-CoA reductase inhibition.  Peptides identified from SGID: CSSSSG, HGAESAGGDT, RTPVP**P**G**L**L; from Alcalase: **DL**VLDVPS, KPSSAAGAVR, TAPHGG**LP**AGDV | [31] |
| Kabuli chickpea protein isolates | Defatted (hexane).  Protein precipitation at isoelectric point. | Alcalase (A) and/or Flavourzyme (F) hydrolysates using 4 combinations:  (1) A;  (2) F;  (3) A1F2 (time 1:2 h);  (4) A2F1 (time 2:1 h).  Common conditions: pH 8, 50–60 °C.  Inact.: 80 °C, 15 min. | Sequential hydrolysis (AF) improved solubility and emulsifying capacities.  Changes were observed in protein secondary structure (36.4-42.8 % β-turn) and profile banding patterns along with a higher degree of hydrolysis (45-50 %) and DPPH· activity (44.3-48.4 %). | [37] |
| Kabuli- (Billy beans, Commercial, Nash, Sierra) and Desi-Myles varieties | Three strategies:  (1) No-treated (raw),  (2) Pre-cooked (boiling water, 15 min),  (3) Cooked (boiling water, 2 h).  Alkaline solubilization (pH 9) and protein precipitation at isoelectric point (pH 4.5). | 1-optimal experimental design (36 runs) for the prediction model: Bromelain was used at E/S (w/w) ratios of 1:10, 1:30 and 1:50, during 0, 30, or 60 min.  Posterior SGID with Pepsin-substrate (1:20 (w/w)), pH 2; and pancreatin-substrate (1:20 (w/w)), pH 7.5, 1.5 h.  Inact.: 79 °C, 10 min. | Raw hydrolysates showed the highest DPP-IV inhibition (IC50: 0.17–3.05 mg soluble protein/mL). α-Amylase inhibitory activity was lower than expected, whereas α-glucosidase inhibition was higher in precooked hydrolysates (53–63%).  39 peptides with antidiabetic potential; Bromelain hydrolysates: albumin EVLSEVSF (MW 908.44 Da, hydrophobic); legumin VVFW (MW 549.29 Da), FDLPAL (MW 674.36 Da). | [19] |
| Chickpea protein (from kabuli Sierra chickpea seeds) | Precooked in boiling water at 98 °C for 15 min.  Alkaline solubilization (pH 9) and protein precipitation at isoelectric point (pH 4.5). | Bromelain (3 units/mg  protein) E/S ratio 1:10; 25 °C, pH 4.5, 60 min.  Inact.: 79 °C for 10 min.  Posterior SGID of pontificated maize tortilla with Pepsin/substrate (1:20 (w/w)), pH 2; and pancreatin/substrate (1:20 (w/w)), pH 7.5, 1.5 h.  Inact.: 79 °C, 10 min. | Maize tortilla fortification (10 % w/w) with Bromelain-CPH enhanced DPP-IV inhibition before (82 %) and after SGID (>50 %); results corroborated by *in silico* analysis.  Main DPP-IV inhibitory peptides identified: FDLPAL, GPGGGGR.  Changes in texture and color were observed and should be evaluated in consumers. | [18] |
| Kabuli (10) and Desi (8) genotypes | Defatted with hexane.  *Albumin extraction*: solubilization in buffer I (10 mM CaCl2, 10 mM MgCl2, 1mM PMSF, pH 8), precipitation in ammonium acetate-methanol (0.1 M) and dialyzed.  *Globulin extraction*: albumin extraction residues in buffer II (0.1 M tris-HCl, pH 8, 100 g/L Na Cl, 1 mM PMSF, 10 mM EDTA), precipitation in ammonium acetate-methanol (0.1 M) and dialyzed.  Preheated at 80 °C for 5 min. | Albumin or globulin fraction suspended 1:10 (w/v).  Alcalase (0.3 U/g protein); 50 °C, pH 8.0, 90 min.  Inact.: 80 °C for 20 min. | CPH showed high antioxidant capacity (ABTS, DPPH) and antidiabetic potential (α-amylase, α-glucosidase, DPP-IV), confirmed by *in silico* and biochemical assays.  FEI, FEL, FIE, FKN, FGKG, MEE from the albumin fraction of genotype ICC3761 demonstrated best antioxidant and antidiabetic potential. | [25] |
| Chickpea (*Cicer arietinum* L.) | Sprouting: seeds were soaked at 30 °C, distilled water sprayed every 3 hours for 3 days.  Defatting with ether.  Protein solubilization (pH 9) and precipitation at isoelectric point (pH 4.5).  Dialyzed for 72 hours, and freeze-dried. | Four enzymatic conditions:  (1) Alcalase pH 9, 45 °C, E/S 3%, 4 h. (2) Papain pH 5.8, 50 °C, E/S 3%, 4 h. (3) Trypsin pH 7.8, 37 °C, E/S 3%, 4 h. (4) Neutrase pH 7.0, 45 °C, E/S 3%, 4 h.  Inact.: boiling 10 min. | The Neutrase-CPH fraction (NF2-4-1) exhibited the strongest antioxidant activity (DPPH IC50: 0.24 mg/mL; hydroxyl radical scavenging IC50: 0.57 mg/mL), the highest degree of hydrolysis (19%), and the richest hydrophobic amino acid composition.  Sequence identification LTEIIP, MW 685.41 Da. | [17] |
| Chickpeas | Defeating with hexane.  Protein solubilization at pH 9.5, and precipitation at isoelectric point (pH 4.5).  Preheating for 5 min at 85 °C. | Two enzymatic conditions:  (1) Alcalase (A) (≥2.4 U/mg), 50 °C, pH 8, 60 min.  (2) Papain (P) (1.5–10 U/mg), 65 °C, pH 6.7, 60 min.  Inact.: 80 °C for 20 min. | A-CPH (10.3 kDa peptides) enhanced foaming properties but showed lower emulsifying capacity. It also demonstrated stronger antioxidant activity (DPPH IC50: 0.32 mg/mL) compared with P-CPH (14.3 kDa peptides) (DPPH IC50: 1.88 mg/mL). | [21] |
| Germinated Kabuli chickpea (Billi bean) | Germination (30 °C, 80% relative humidity, for 2, 4 and 6 days).  Defatted with hexane.  Protein solubilization at pH 9.5, and precipitation at isoelectric point (pH 4.5). | Two enzymatic conditions from  CPI 1:40 (w/v).  (1) Papain: 25 °C, pH 6.2.  (2) Ficin: 37 °C, pH 7.  With conditions: E/S (w/w) ratios of 1:10, 1:30 and 1:50. 0, 30, 60 min.  Inact.: 80 °C for 5 min.  Post-hydrolysis SGID: Pepsin/Pancreatin method. | Ficin-CPH showed higher DPP-IV inhibitory activity than Papain-CPH.  Identified peptides: SPGAGKG (DPP-IV IC50: 1.78 mg/mL); GLAR (DPP-IV IC50: 8.74 mg/mL); STSA; showed potential DPP-IV and ACE inhibitory activities. | [12] |
| Legumin and provicilin sequences from Chickpea | *In silico* protein fraction selection. | *In silico* hydrolysis conducted with BIOPEP-UWM database using 5 single-enzymes:  (1) Pepsin (pH 1.3, EC 3.4.23.1).  (2) Trypsin (EC 3.4.21.4).  (3) Chymotrypsin (EC 3.4.21.1).  (4) Papain (EC 3.4.22.2).  (5) Alcalase (EC 3.4.21.62). | Molecular docking and ADMET analyses revealed strong ACE-I binding of peptides through hydrogen bonds and salt bridges.  A total of 216 legumin-derived and 165 provicilin-derived ACE-I inhibitory peptides were predicted, with over 57 % featuring hydrophobic residues (Pro, Leu, Tyr) at the C-terminus. | [42] |
| Green pea (*Pisum sativum*), chickpea (*Cicer Arietinum*), and navy bean  (*Phaseolus vulgaris*) | Soaked.  Cooked treatments (conventional (CC), pressure (PC) and microwaved (MC) cooked). | SGID using the INFOGEST method. | α-Amylase inhibition activity by <3 kDa fractions of green pea, chickpea and navy bean, after different types of household cooking (conventional, pressure, and microwave cooking) and GID.  205 peptides identified including 43 with *in silico* antidiabetic activity. | [28] |
| Chickpea (*C. arietinum* L.) seeds | Defatted (extraction gasoline).  Osborne protein fractionation. | 1:100 (w/v) of each fraction in 0.05 M of Na_3_PO_4_ were treated:  Trypsin 1:50 (w/w) E/S ratio: 37 °C, pH 8.0, 7 h.  Inact.: 95 °C for 10 min.  CPHs 10 mg/mL were heated 50 °C and pH 6 adding ZnSO_4_ (10:1 (w/w)). | CPH showed strong zinc-binding capacity (observed in both chelating and molecular docking assays) and significant ACE2 inhibitory activity.  Identified peptide HKERVQLHIIPTAVGK (MW 1826 Da) with highest Zn binding and ACE2 inhibition activities. | [46] |
| Kabuli chickpea | Protein solubilization at pH 11, and precipitation at isoelectric point (pH 4).  Defatted with hexane.  Preheated in a water bath at 80 °C for 20 min. | Four enzymatic conditions:  (1) Alcalase: 55 °C, pH 8.5.  (2) Neutrase: 45 °C, pH 7.0.  (3) Protamex: 55 °C, pH 8.0.  (4) Papain: 55 °C, pH 7.0.  E/S: 8000 U/g.  Inact.: 90 °C for 10 min.  Post-hydrolysis INFOGEST method to SGID. | Alcalase-digested hydrolysate exhibited the highest ADH activation capacity (IC50: 0.05 mg/mL).  *In silico* analysis identified four peptides as ADH activators (ILPHF, MFPHLPSF, LMLPHF and FDLPALRF). | [27] |
| Germinated chickpea protein | Germination.  Defatted.  Protein solubilization at pH 9, and precipitation at isoelectric point (pH 4.3). | Sequential hydrolysis from  CPI 1:50 (g/v): Alkaline protease, 2% (w/w), 1 h, pH 10, 37 °C.  Inact.: boiling 10 min.  Post-hydrolysis SGID: Pepsin/Trypsin. | CPH demonstrated antioxidant capacity (DPPH, Hydroxyl radical scavenging activity, FRAP), and protection against AAPH-induced erythrocyte hemolysis, lipid peroxidation and hemoglobin oxidation.  Structural shifts ↑α-helix (26 %) and β-turn (43 %), ↓β-sheet (9.4 %). | [13] |
| *C. arietinum* L., *C. reticulatum,* *C. echinospermum*, *C. pinnatifidum*, *C. judaicum*, and *C. cuneatum* seeds | Protein extraction as reported (Yust et al., 2003). | Six enzymatic conditions during 120 min:  (1) Alcalase: Tris-HCl, pH 8, 55 °C, 1:50 E/S, 150 rpm.  (2) Chymotrypsin: phosphate buffer, pH 6.8, 50 °C, 1:50 E/S, 150 rpm.  (3) Flavourzyme: Tris-HCl, pH 8, 55 °C, 1:50 E/S, 150 rpm.  (4) Papain: phosphate buffer, pH 6.5, 65 °C, 1:50 E/S, 150 rpm.  (5) Pepsin: KCl-HCl, pH 1.5, 37 °C, 1:50 E/S, 150 rpm.  (6) Trypsin: Tris-HCl, pH 8, 37 °C, 1:50 E/S, 150 rpm.  Inact.: boiling 10 min. | Alcalase-CPHs (ranged 3.5 to 17 kDa) showed the strongest bioactivities:  DPPH 72.3% vs Papain-CPH 42.3%; β-carotene 74.1% vs Papain-CPH 46%; FRAP 2.1 vs Papain-CPH 0.9; Fe²⁺ chelation 66.2% vs Chymotrypsin-CPH 42.1%; ACE inhibition 66.1% vs Papain-CPH 42.9% (and Pepsin-CPH 41.1%).  Alcalase-CPHs also showed slight HYA inhibition (IC50: 57.2 µg/mL) and LOX inhibition (IC50: 32.6 µg/mL). | [43] |
| Chickpea seeds | Defatted with ether.  Protein solubilization at pH 8, and precipitation at isoelectric point (pH 5.5).  Preheating: 80°C, 20 min. | Orthogonal design: CPI 1:20 (w/v); papain + neutral protease, E/S 8%, 50–55 °C, pH 6.8–7.0, 3 h.  Inact.: 90–95 °C for 10 min.  Chelation: preheating 10 min with 0.3 g anhydrous CaCl₂, then mixing with CPH for 2 min. | Optimized hydrolysis (1:15 ratio, 10 min, 20% E/S, 60 °C) produced CPH with strong antioxidant activity (•OH: 49.4%, O₂⁻: 63.6%, DPPH: 66.6%, H₂O₂: 57.6%, reducing power: 0.753). Optimum Ca-chelation (7:1 ratio, 40 °C, pH 7.0, 20 min) reached 20.9% chelation rate. | [26] |
| Desi-type chickpea (Cicer arietinum L.), Brazilian cultivar GB Cappuccino | Whole seeds were ground and passed through a 45-mesh sieve.  Globulins were extracted three times with 0.5 M NaCl (1:10, w/v; 30 min). Combined supernatants were dialyzed against water for 24 h at 5 °C.  Globulins were precipitated by centrifugation (7,000 × g, 30 min, 5 °C) and freeze-dried.  No defatting treatment was reported. | Post-extraction SGID:  Pepsin: 50 mg protein in 7.5 mL KCl–HCl buffer (0.1 M, pH 1.5), 0.75 mg pepsin, 37 °C, 3 h.  Pancreatin: addition of 7.5 mL sodium phosphate buffer (0.2 M, pH 8.0) containing 2 mg pancreatin, 37 °C, up to 24 h.  Inact.: boiling for 5 min. | Pepsin caused only partial globulin hydrolysis, whereas subsequent pancreatin digestion considerably increased peptide release.  ABTS antioxidant activity increased from approximately 650 nmol Trolox equivalents/mg after pepsin hydrolysis to approximately 1,400 nmol Trolox equivalents/mg after pepsin–pancreatin digestion.  ACE inhibition was not detected after pepsin digestion alone. The pepsin–pancreatin hydrolysate showed ACE-inhibitory activity (IC₅₀: approximately 1.138 mg/mL; 694.55 nmol/mL). | [RS10] |
| Kabuli chickpea (Cicer arietinum L.), cultivar Blanco Sinaloa: non-germinated seeds (Raw), non-biofortified sprouts (G), and sodium-selenite-biofortified sprouts (GSe) | Germination: 22 °C, 80% relative humidity, 48 h, with irrigation every 4 h. GSe sprouts were biofortified with sodium selenite (24 mg/L).  Seeds/sprouts were dried at 55 °C for 24 h, ground, and passed through a 180-µm sieve.  Defatted with hexane (1:4, w/v), 50 °C, 200 rpm, 12 h; solvent replaced after 6 h.  Protein solubilization at pH 8.5 for 2 h and precipitation at the isoelectric point (pH 4.5) for 2 h. Protein extracts were freeze-dried. | Flavourzyme® (≥500 U/g): pH 7.0, 50 °C, 200 rpm.  E/S ratios and times: 2 or 4% for 2 h; 3% for 4 h; and 2 or 4% for 6 h.  Protein extract/water: 1.25 g/50 mL. Preheating at 50 °C for 15 min.  Inact.: 90 °C for 15 min. | Hydrolysis produced an approximately threefold increase in α-amino nitrogen after 6 h, although no significant difference was observed between 4 and 6 h.  At 4% E/S and 6 h, GSe hydrolysates increased umami-associated Asp and Glu contents by 40% and 51.5% compared with G and Raw, respectively.  DPPH scavenging activity was 19% and 25% higher in GSe and G hydrolysates, respectively, than in Raw hydrolysates. GSe hydrolysates showed the highest ORAC activity, with a 21% increase at 4% E/S and 6 h. Maximum ABTS activity was also observed at 4% E/S and 6 h, without significant differences among Raw, G, and GSe. | [[RS9]](https://doi.org/10.1016/j.fufo.2026.101135) |
| Kabuli chickpea (Cicer arietinum L.) protein isolate | Protein isolate obtained according to the patented procedure cited by the authors (Patent No. 2803851).  Defatting, solubilization, and precipitation conditions were not described in the article. | Hydrolysis to 10% degree of hydrolysis using:  Bromelain: pH 4.5, 45 °C. Papain: pH 6.2, 45 °C. Pepsin: pH 2.6, 37 °C. Trypsin: pH 7.5, 37 °C. Protoferm FP: pH 3.5, 45 °C. Alcalase 2.4 L FG: pH 7.0, 45 °C.  E/S ratios: 0.5, 1, 1.5, or 2%. Alcalase at 2% reached 10% hydrolysis in 20 min. Protoferm FP required 4% E/S and 120 min.  Samples used for characterization: 1.5% E/S for plant- and animal-derived enzymes and 2% for microbial enzymes.  Post-hydrolysis centrifugation: 4,000 rpm, 15 min. Enzyme-inactivation conditions were not reported. | Pepsin- and Protoferm FP-treated hydrolysates exhibited the highest antioxidant activity, increasing ORAC by 303.7% and 217.7%, respectively, compared with the native isolate (1,447.19 and 1,037.37, respectively).  Pepsin hydrolysate showed the highest DPPH radical-scavenging activity, while pepsin, papain, and Protoferm FP hydrolysates exhibited the greatest FRAP activity.  Trypsin and papain degraded more than 55% of the original protein. Peptide profiling and in silico analysis predicted antithrombotic, antitumor, antibacterial, antioxidant, and antiamnesic activities. | [[RS8]](https://doi.org/10.21603/2308-4057-2026-1-666) |

CPI (chickpea protein isolate), CPH (chickpea protein hydrolysate), SGID (simulated gastrointestinal digestion), w/v (weight/volume), LAPU (leucine aminopeptidase units), E/S (enzyme/substrate ratio), FRAP (ferric reducing antioxidant power), ACE (angiotensin I–converting enzyme), DPPH (2,2-diphenyl-1-picrylhydrazyl), ABTS (2,2′-azinobis(3-ethylbenzothiazoline-6-sulfonic acid)), ORAC (oxygen radical absorbance capacity), DPP-IV (dipeptidyl peptidase IV), w/o (water-in-oil), w/w (weight/weight), TBARS (thiobarbituric acid reactive substances), ADMET (absorption, distribution, metabolism, excretion, toxicity), ADH (alcohol dehydrogenase), HYA (hyaluronidase), LOX (lipoxygenase), HHP (high hydrostatic pressure), RH (relative humidity), Inact. (Inactivation).

# Table S3. Description of cellular and *in vivo* studies on chickpea protein hydrolysates (CPHs): raw materials, pre-treatments, hydrolysis conditions, cellular line or animal model, and key findings.

| **Raw Material** | **Pre-treatments, defatting and precipitation methods** | **Hydrolysis conditions and other treatments (digestions or incubations)** | **Cellular line/animal model** | **Main Results** | **Source** |
| --- | --- | --- | --- | --- | --- |
| Chickpea flour | Solubilization (pH 10.5) and protein precipitation at isoelectric point (pH 4.3). | CPI 2 % (w/v) treated with sequential action:  -Pepsin: 37 °C; pH 2.5; 180 min.  -Pancreatin: 37 °C; pH 7.5. E/S ratio 1/20 (w/w), 180 min.  Inact.: 80 °C, 20 min. | Caco-2 and THP-1 cell lines (co-cultured). | The decrease in Caco-2 cell viability (MTT), together with the increase in THP-1 cells, suggests that the generated peptides may exert anticancer effects in colon cells with an immunomodulatory role. | [71] |
| Chickpea seeds | Defatted (hexane).  Solubilization (pH 10 in 0.25 % Na_2_SO_3_ (w/v)) and protein precipitation at isoelectric point (pH 4.3).  Spray-dried. | Sequential hydrolysis of CPI 5 % (w/v) with:  -Pepsin: 37 °C; pH 2.5; 180 min.  -Pancreatin: 37 °C; pH 7.5. E/S ratio 1/20 (w/w), 180 min.  Inact.: 80 °C, 20 min. | Caco-2 cell. | Fractions (F1, F2, F3) showed high metal-chelating capacity (Cu^2+^ and Fe^2+^) and lipid peroxidation prevention (β-carotene, TBARS).  Subfractions demonstrated high antioxidant activities in *ex vivo* assays (FRAP, FRSA) and cellular antioxidant activity in Caco-2 cell–based assays.  Legumin peptides with high antioxidant activities: A**L**E**P**DHR, TETWNPNH**P**E**L**, **F**V**P**H, SAEHGSLH | [38–41] |
| Chickpea seeds | Defatted with hexane.  Alkaline solubilization (pH 8) and protein precipitation at isoelectric point (pH 4.3). | CPI 2 % (w/v).  Alcalase 2.4 L FG (0.4 mL/L), 50 °C, pH 8, 40 min.  Inact.: 95 °C, 10 min. | Caco-2 y HT-29 (0.05-0.5 mg/mL of peptides). | Antioxidant peptide efficiently quenched: the free radical of DPPH, superoxide, and hydroxyl, metal ion (Cu^2+^ (76.92 %) and Fe^2+^ (63.08 %) at 50 µg/mL. Frac.7 showed greater linoleic acid autoxidation inhibition than that of a-tocopherol (88.81 %).  Fra.IV exhibited the highest antioxidant activities. From this fraction, Fra.7 was isolated, from which the peptide NRYHE (MW 717.37 Da).  The pentapeptide protected human intestinal cells from H_2_O_2_-induced oxidative damage, stimulated antioxidant enzymes, and activated genes regulated by the Nrf2–ARE pathway. | [57, 58, 73] |
| Chickpea seeds | Defatted chickpea meal.  Solubilization with distilled water, precipitation at pH 4.3, discard the precipitate, new precipitation at pH 3.7 for albumin extraction. | Sequential hydrolysis of CPI with:  -Alcalase at E/S ratio of 0.3 AU/g, 50 °C, pH 8.0, 1 h.  -Flavourzyme at E/S ratio of 50 LAPU/g, 50 °C, pH 7.0, 2 h.  Inact.: 80 °C for 10 min. | KM mice with normal or high fat diet (N=10 per group): control, CPH (50, 100 or 150 mg/kg bw).  H-22 cells. | *In vivo* evaluation in tumor-bearing KM mice and H-22 hepatic carcinoma cells showed reduced lipid levels and decreased tumor volume in KM mice.  Serum analysis also revealed a marked increase in SOD activity and a decrease in MDA levels. | [68] |
| Chickpea (*Cicer arietinum* L.) seeds | Boiled (60 min, 100 °C) and raw chickpea seeds.  Protein precipitation at isoelectric point (Girón-Calle et al., 2010). | SGID similar to INFOGEST. | BJ (ATCC CRL-2522). | CPHs were fractionated into three peptide fractions: <3.5, 3.5–7.0, and <7.0 kDa.  Boiled-SGID fractions showed ABTS activity (IC50 41–44.2 ± 2.1 μg/mL). The <3.5 kDa fraction exhibited lower Fe²⁺ but higher Cu²⁺ chelating activity (IC50: 56.6 μg/mL) and a FRAP value of 0.362 A700/μg mL−1.  Fibroblast growth stimulation activity was also observed. | [29] |
| Desi-green chickpea (ICC5613)  Kabuli-type, cream (Blanco Sinaloa) | Soaked seeds were germinated/sprouted during 5 days in darkness conditions at 24 °C and 80 % RH.  Defatted with hexane.  Protein extraction at pH 8.5 and precipitation at isoelectric point (pH 4.5). | Cooked and germinated chickpeas were digested in SGID using method (Ortega et al., 2009).  Freeze dried samples were extracted in 80 % aqueous ethanol. | RAW 264.7 cells (CPI and digests 0.5-5 mg/mL). | Anti-inflammatory potential in LPS-activated RAW macrophages (increased cell viability and nitric oxide inhibition).  Peptide identification: LHQNIGSSSSPDIYNPQAGR,  LHQNIGSSSSPDIYNPQAGRIK, QQSQETDVIVK, VLLEEQEQKPK, DVGQKTKEVGQDIQAK. | [14] |
| Chickpea seeds (45 accessions comprising Desi, Kabuli and wild) | Defatted with hexane.  Alkaline extraction and isoelectric precipitation. | Alcalase 2.4 L (E/S  2% (w/v)), 1 h, 50 °C, pH 8.0.  Inact.: 85 °C for 15 min; lyophilized and filtered (0.45 μm).  Post-hydrolysis SGID with Trypsin-Chymotrypsin method. | MCF-7 and MDA-MB-231 cells (treated with BDN-9-3). | *In vitro* digestibility ranged: 56-75 %.  Peptides from 45 chickpea accessions showed varying ACE-I inhibition (IC50: 22.43–47.48 mg/mL); BDN-9-3 had strongest effect.  BDN-9-3 showed antiproliferative activity (SRB assay) on MCF-7 (IC50: 0.60 mg/mL), and on MDA-MB-231 (IC50: 0.63 mg/mL). | [49] |
| *Cicer arietinum*, and *Cicer reticulatum* | Defatted with hexane.  Protein precipitation at isoelectric point (Pedroche et al., 2002). | Two hydrolysis conditions:  (1) Alcalase (2% w/v), 50 °C, pH 8, 60 min.  (2) Flavourzyme (2% w/v), 50 °C, pH 7, 100 min.  Heat inactivation. | Ishikawa and MCF-7 cells. | Alcalase-CPHs exhibit stronger DPPH (55-69 %) and ACE-I inhibitory (IC50: 0.182 mg/mL) activities.  CPHs exhibited antiproliferative effects (SRB assay) in Ishikawa (IC50 of *C. arietinum* and *C. reticulatum* were 0.36 and 0.29 mg/mL, respectively) and MCF-7 (IC50 of *C. arietinum* and *C. reticulatum* were 0.47 and 0.41 mg/mL, respectively) cell lines. | [62] |
| Chickpea seeds | Defatted with hexane.  Protein extraction at pH 9 and precipitation at isoelectric point (pH 4.3). | CPI 5 % solution. Three hydrolysis conditions:  (1) Pepsin (PEP) (1:10 E/S ratio): 37 °C, pH 2.0, 30, 60, 90, and 120 min.  (2) Pancreatin (PAN) (1:10 E/S ratio), 37 °C, pH 7.0, 30, 60, 90, and 120 min.  (3) subsequential PEP-PAN (1:10 E/S ratio), 37 °C, pH 2.0-7.0, 15, 30, 45, 60 min (for each enzyme).  Inact.: 80 °C for 20 min. | Male ICR mice (12×6); diet 50% normo-caloric and 50% hypercaloric; CPH 10, 20, 30 mg/kg bw; AOM as tumor inducer. | ABTS values of CPHs ranged from 18.9 to 24.8% inhibition.  *In vivo* assessment demonstrated protective effects against colon cancer under a hypercaloric diet, along with reductions in total cholesterol and LDL following Pepsin-Pancreatin-CPH consumption. | [48] |
| Chickpea | Defatted.  Albumin, globulin, and glutelin (precipitation).  Protein denaturalization (TRIS, urea, and 1.4-dithiothreitol) (1h, 30 min). | Enzymatic hydrolysis (enzyme/protein 1:50) was performed for 16 h at 25 °C using Chymotrypsin from bovine pancreas. | Caco-2 cells. | Identified peptides (Leg1: RIKTVTS**FDLPAL**RFLKL; Leg2: RIKTVTS**FDLPAL**RWLKL) showed antibacterial, antifungal, and antioxidant (DPPH, FRAP) activities. Caco-2 cell assays (MTT) confirmed no cytotoxicity. | [63–65] |
| Chickpea seeds | Defatted (with petroleum ether).  Osborne albumin fractionation. | Subsequential hydrolysis of CPI:  (1) Papain: pH: 6.0, 12 h.  (2) Trypsin: pH 8.0, 12 h.  (3) Alcalase: pH: 10, 12 h.  Inact.: 95 °C for 10 min.  Synthesis of vanadium-CPH complexes at pH 4, temp 40 °C and 5 mg/mL of VOSO_4_. | A549, Hela 229, and T47D cell lines. | Vanadium-CPH exhibited inhibitory effects on A549 lung cancer cells in MTT assays (IC50: 14.4 µg/mL).  *In silico* analysis revealed bio-penetrating peptides likely contributing to the anticancer activity.  Identified peptides: ESEFDFR; FDDEKEFR; EENPEEAR; SLSDFR; DFSER; YPGSC; AFSD; MW 437 to 1864 Da | [56] |
| Pigeon pea, chickpea and lentil seeds | Drying overnight with vacuum conditions.  Defatted with hexane.  Protein solubilization at pH 10, and precipitation at pH 5. | Two enzymatic conditions:  (1) Alcalase E/S 1% (w/v), pH 7.5, 30 min.  (2) Bromelain E/S 1% (w/w), pH 6.5, 30 min.  Inact.: boiling for 10 min. | RAW 264.7 (Treatment with 1 mg/mL). | Hydrolysates (<5 kDa profile) exhibited antioxidant activity (DPPH, ORAC, NO radicals) and anti-inflammatory effects through inhibition of NO production in lipopolysaccharide-induced macrophages.  Although lower activity was observed in biochemical assays, no differences were detected in cell-based assays. | [67] |
| Kabuli chickpea (ICC 3421) | Flour Extrusion (at 150 °C and screw speed of 240 rpm).  Defatted with hexane.  Alkaline solubilization (pH 8.5) and protein precipitation at isoelectric point (pH 4.5). | Extruded and unextruded flour were digested in SGID using method (Ortega et al., 2009) similar to Milán-Noris et al 2018. | Male SHRs with commercial diet supplemented with CPHs (1.2 g/kg bw). | ACE-I inhibitory activity: IC50 values of 0.32 mg/mL for extruded and 0.28 mg/mL for unextruded samples.  *In vivo*, the extruded hydrolysate produced a stronger antihypertensive effect than the unextruded counterpart in male SHRs, suggesting a different peptide profile. | [33] |
| Desi chickpea (ICC3761) | Defatted with hexane.  Albumin solubilization (pH 8) and washed with methanol and acetone.  Preheated at 80 °C for 5 min. | Two enzymatic conditions:  (1) Alcalase (0.3 AU/g protein): 50 °C, pH 8.0, 180 min.  (2) SGID using Pepsin-Pancreatin method. | BALB/c mice. | Alcalase- and SGID-CPHs showed strong antioxidant activity (ABTS, DPPH).  *In vivo* experiment improved ALP, MDA, GSH, hypoglycemic and antihyperglycemic effects; only SGID reduced postprandial hyperglycemia; both showed anti-inflammatory activity. | [24] |
| Kabuli chickpea seeds (Blanco Sinaloa) | Germination with 2 mg Na_2_SeO_3_/100 g of seeds for 6 h.  Defatted with hexane.  Protein solubilization at pH 8.5 and precipitation at isoelectric point (pH 4.5).  Osborne fractionation. | Three enzymatic conditions:  (1) Alcalase: 60 °C, pH 8.0.  (2) Pepsin: 37 °C, pH 2.2.  (3) Trypsin: 37 °C, pH 8.0.  E/S ratio 4% w/w, 30, 60, 120 min.  Inact.: 90 C for 5 min. | HDFa cells. | Pepsin and Trypsin hydrolysates had the highest antioxidant potential (DPPH, ORAC, superoxide scavenging) and Se stability.  Hydrolysates <10 kDa inhibited collagen degradation after UVA radiation in HDFa cells, indicating potential cosmetic applications. | [15, 16] |
| Germinated Kabuli chickpea (Billi bean) | Methodology from (Chandrasekaran & Gonzalez de Mejia, 2022). | CPI was dissolved 1:40.  Ficin at 1:10 E/S ratio, 37 °C, pH 7, 30 min. | Caco-2 cells (CPH: 0.5-2.5 mM). | Biochemical and molecular docking analyses indicated that CPH inhibited DPP-IV and α-glucosidase. Peptides: VVFW, GEAGR, and FDLPAL from legumin.  Cell-based assays confirmed DPP-IV inhibition (IC50: 2.1 mM), dose-dependent glucose uptake inhibition (IC50: 2.0 mM), and suppression of GLUT2 and T2R14 gene expression in Caco-2 cells. | [11] |
| Kabuli variety chickpea | Protein solubilization at pH 11, and precipitation at isoelectric point (pH 4).  Defatted with hexane.  Preheated in a water bath at 80 °C for 20 min. | Hydrolysis using 1 or combinations of 2 enzymes after heat Inact.:  Neutrase: pH 7.0, 45 °C.  Protamex: pH 8.0, 55 °C.  Alcalase: pH 8.5, 55 °C.  Papain: pH 7.0, 55 °C.  E/S ratio 8000 U/g.  Inact.: 80 °C for 20 min.  Post-hydrolysis SGID (INFOGEST). | Caco-2 cells. | Post-hydrolysis and SGID identified 4,847 peptides.  *In silico* prediction indicated DPP-IV inhibitory peptides (AAWPGHPEF, LAFP, IAIPPGIPYW, PPGIPYW); two were validated in cellular model, demonstrating non cytotoxic effect (MTT) and significant DPP-IV inhibition (IC50: 138-270 µM). | [52] |
| Kabuli chickpea (Blanco Sinaloa 92) | Defatted with acetone.  Protein solubilization at pH 8.5, and precipitation at isoelectric point (pH 4.5). | Hydrolysis with Alcalase (0.2, 0.3, or 0.4 (U/g) E/S ratio), 0.5, 1.5, or 2.5 h; at 40, 50, or 60°C, pH 8.5.  Inact.: 85 °C for 15 min. | Male SHRs (CPH: 50 mg/g bw; BIS-TRIS Propane buffer and captopril as controls), N=7 per group. | The optimized CPH (0.5 h, E/S 0.25 U/g, 40 °C) showed higher ACE-inhibitory activity (56.2 %).  *In vivo* experiment, CPH lowered blood pressure by −24.3 mmHg (1 h) and −47.3 mmHg (7 h) post-supplementation. | [60] |
| Commercial concentrate of kabuli chickpea |  | At pilot scale (CPI 1:10 (w/v)):  Hydrolysis with Bioprotease at 30 min; pH 8; E/S ratio 0.3 (U/g protein); 50 °C.  Inact.: 80 °C for 20 min (H30BIO).  Post-hydrolysis Pepsin/Pancreatin SGID. | THP-1 cell line (CPH: 0.05-0.5 mM). | The H30BIO hydrolysate (<2.88 kDa) exhibited antioxidant activity (DPPH, FRAP) and high digestibility.  In a cellular model, it reduced IL-1β, NF-κB, and NLRP3 expression, restored mitochondrial SOD, and suggested potential mitochondrial protection through ROS/NO pathways.  It also showed synergistic activity with vitamin D in the same model. | [34, 69, 70] |
| Chickpea protein concentrate (60 % protein) | Protein solubilization at pH 9.5, and precipitation at isoelectric point (pH 4.5). | Sequential digestion:  (1) Alcalase at E/S ratio of 0.3 AU/g, 50 °C, pH 8.0, 1 h.  (2) Flavourzyme at E/S ratio of 50 LAPU/g, 50 °C, pH 7.0, 2 h.  Inact.: 80 °C for 10 min (Kou et al., 2013). | C57BL/6J mouse (placebo group, CPH 400 mg/kg bw, and CPH 800 mg/kg bw), N=13 per group. | Identified peptides: DLPAL (527.29 Da); STTGVG (545.54 Da); GKSPGAGGGSL (886.45 Da); *in silico* predicted as ACE and DPP-IV inhibitors using BIOPEP and PepDraw.  *In vivo* results: CPH (800 mg/kg) reduced body weight by 24.8% and showed the lowest NAFLD score.  CPH (400 mg/kg) downregulated genes involved in fatty acid uptake (Slc27a1), adipokine signaling (Tnfrsf1b) and insulin signaling (Pik3r1, Ikbkb, Map2k1). | [61] |
| Chickpea powder (63% protein) |  | Sequential digestion:  (1) Pepsin pH 2.0, 37 °C, 2 h, pH 8.  (2) Trypsin + Chymotrypsin 37 °C, 2 h.  Inact.: 100 °C, 10 min. | C2C12 cell line. | CPH and CPP737 peptide induced glucose uptake through AMPK activation and GLUT4 membrane translocation in C2C12 myotubes.  Identified peptides: REGDIIAVPTGVVF CPP737, LAQALNTDEDTAKKL, derived from a legumin A like protein; and LANRDDNEDLRVL and SSGDVVVIPASHPF from vicilin-like protein. | [74] |
| Kabuli chickpea (Cicer arietinum L.) | Seeds ground into flour and stored at 4 °C.  CPI prepared from chickpea flour.  Ultrasound pre-treatment: CPI/water 1:11 (w/v), 20 kHz, 50% amplitude, 50 °C, 15 min (10 s on/2 s off). | Alcalase: pH 8.0, 50 °C, 1% E/S, 60 min.  Ultrafiltration (<3 kDa fraction, UCPH-1) followed by FPLC; F6 identified as MLRVYLR.  Post-hydrolysis SGID. | Artemia salina nauplii (brine shrimp lethality assay).  In vitro antioxidant and ACE-inhibitory assays and in silico molecular docking. | Ultrasound increased the degree of hydrolysis to 70.63% and enhanced the antioxidant activity of UCPH.  UCPH-1 and MLRVYLR inhibited lipid peroxidation. MLRVYLR was non-toxic to A. salina.  UCPH-1 and F6/MLRVYLR showed 85.82% and 95.76% ACE inhibition, respectively (IC₅₀: 0.35 and 0.20 mg/mL). MLRVYLR retained 91.1% activity after SGID and behaved as a competitive ACE inhibitor. | [RS2, RS3, RS4] |
| Chickpea seed waste | Washed and dried at 60 °C for 24 h, followed by grinding.  Protein solubilization at pH 8.8 for 2 h and precipitation at the isoelectric point (pH 4.5) overnight at 4 °C.  Dialysis and lyophilization. | Trypsin: pH 8.0, 37 °C, E/S ratio 1:100 (w/w; 4,000 U/g), 40 min.  Inact.: 95 °C for 15 min. | HepG2 human hepatocellular carcinoma cells.  MCF-7 human breast adenocarcinoma cells.  Listeria innocua and Escherichia coli. | Chickpea waste peptides inhibited HepG2 and MCF-7 cell proliferation by 83% and 86%, respectively, at 100 µg/mL (IC₅₀: approximately 20 µg/mL).  Peptides also showed antibacterial activity and were subsequently incorporated into a functional coating for probiotic-enriched Ras cheese. | [[RS6](https://doi.org/10.1016/j.rineng.2025.105352)] |
| Kabuli chickpea (Cicer arietinum L.), cultivar Blanco Sinaloa 92 | Defatted with acetone (1:4, w/v; 500 rpm, 4 h).  Protein solubilization at pH 8.5 for 2 h and precipitation at the isoelectric point (pH 4.5).  Lyophilized and stored at −20 °C. | Optimized hydrolysis with Alcalase: pH 8.5, 40 °C, E/S ratio 0.254 U/g, 30 min.  Inact.: 85 °C for 15 min. | Male spontaneously hypertensive rats (SHRs).  OCPH: 50 mg/kg bw/day for 5 weeks; vehicle as control. N = 7 per group. | OCPH reduced systolic, diastolic, and mean blood pressure from day 5, with a maximum systolic reduction of 39.80 mmHg.  Renal ACE2 and Mas1 expression increased by 67.3% and 61.1%, respectively, whereas ACE1, renin, and AT1R remained unchanged, suggesting activation of the ACE2/Ang-(1–7)/Mas1 axis. | [[RS5](https://doi.org/10.3390/foods14203537)] |
| Desi chickpea (Cicer arietinum L.), genotype ICC 3761 | Defatted chickpea flour dispersed in water at pH 8.0 for 2 h.  Albumin-containing supernatant recovered by centrifugation and lyophilized.  Proteins washed sequentially with methanol and acetone. | Pepsin: pH 2.0, 37 °C, 90 min.  Pancreatin: pH 8.0, 37 °C, 90 min.  Inact.: 80 °C for 20 min.  Filtration through 0.45 µm and ultrafiltration through a 10 kDa membrane. | Male Wistar rats with high-fat diet-, sucrose-, and streptozotocin-induced type 2 diabetes-like condition.  Five groups, N = 6: healthy control, diabetic control, metformin (500 mg/kg), CAH (200 mg/kg), and CAH (400 mg/kg). Oral treatment for 30 days. | CAH reduced fasting blood glucose and improved sucrose tolerance, lipid profile, renal and hepatic biochemical markers, antioxidant status, and pancreatic histology.  CAH decreased hepatic PEPCK and G6Pase activities and increased G6PD activity. Exploratory immunoblotting indicated modulation of AMPK and AKT phosphorylation. | [[RS7](https://doi.org/10.1111/1750-3841.71178)] |

CPI (chickpea protein isolate), CPH (chickpea protein hydrolysate), SGID (simulated gastrointestinal digestion), w/v (weight/volume), LAPU (leucine aminopeptidase units), E/S (enzyme/substrate ratio), FRAP (ferric reducing antioxidant power), ACE (angiotensin I–converting enzyme), DPPH (2,2-diphenyl-1-picrylhydrazyl), ABTS (2,2′-azinobis(3-ethylbenzothiazoline-6-sulfonic acid)), ORAC (oxygen radical absorbance capacity), DPP-IV (dipeptidyl peptidase IV), w/o (water-in-oil), w/w (weight/weight), TBARS (thiobarbituric acid reactive substances), ADMET (absorption, distribution, metabolism, excretion, toxicity), ADH (alcohol dehydrogenase), HYA (hyaluronidase), LOX (lipoxygenase), HHP (high hydrostatic pressure), RH (relative humidity), AOM (azoxymethane), NAFLD (Non-alcoholic fatty liver disease), MTT (3-(4,5-dimethylthiazol-2-yl)-2,5-diphenyltetrazolium bromide), ALP (Alkaline phosphatase), MDA (Malondialdehyde), GSH (Reduced glutathione), Inact. (Inactivation).

# Table S4. Proximal Macronutrient Composition of Raw Chickpeas.

| **Nutrient** | **Content ^a^** | **Content ^b^** | **DRV ^c^** |
| --- | --- | --- | --- |
| Energy (kcal) | 365.0 | 336.0 | 2000.0 |
| Fat (g) | 4.0 – 10.0 | 6.3 | 78.0 |
| Protein (g) | 15 – 40 | 19.3 | 50.0 |
| Carbohydrates (g) ^d^ | 62.0 – 70.0 | 64.1 | 275.0 |
| Fiber (g)  Soluble  Insoluble | 18.0 –22.0  4.0 – 8.0  10.0 – 18.0 | 14.9 | 28.0 |
| Humidity (g) | 7.3 |  |  |

The values are presented in grams per 100 grams of dry product, except Energy (kcal), according to:

^a^ [12]

^b^ [S3].

^c^ Dietary Reference Values (DRV) are based on a caloric intake of 2000 kcal for adults [S4].

^d^ Carbohydrate value including dietary fiber.

# Table S5. Vitamin and Mineral Content of Raw Chickpeas.

| **Micronutrient** | **Content^a^** | **DRV^b^** |
| --- | --- | --- |
| Vitamin A retinol equivalents from retinoids and carotenoids activities (µg) ^c^ | 21.50 | 650-750 |
| Vitamin E, alpha-tocopherol equivalents from vitamin E vitamers activities (mg) | 3.10 | 11-13 |
| Vitamin K (µg) | 9.00 | 65-90 |
| Folic acid (Folates, B9) (µg) | 185.00 | 330.00 |
| Niacin equivalents (B3) (mg) | 1.70 | 14-17 |
| Riboflavin (B2) (mg) | 0.14 | 1.2-1.5 |
| Thiamine (B1) (mg) | 0.45 | 1-1.2 |
| Pyridoxine (B6) (mg) | 0.15 | 1.2-1.7 |
| Ascorbic acid (vitamin C) (mg) | 4.10 | 75.00 |
| Calcium (mg) | 143.00 | 950-1150 |
| Copper (mg) | 0.66 | 1-1.3 |
| Iron (mg) | 6.80 | 9-18 |
| Potassium (mg) | 1000.00 | 3500.00 |
| Magnesium (mg) | 122.00 | 280-350 |
| Manganese (mg) | 21.31 | 3.00 |
| Sodium (mg) | 30.00 | 1500.00 |
| Phosphorus (mg) | 310.00 | 700-800 |
| Iodure (µg) | 1.50 | 150.00 |
| Selenium (µg) | 2.00 | 55-70 |
| Zinc (mg) | 2.00 | 7-11 |

^a^ The values are presented in mg or µg per 100 grams of dry product [S3].

^b^ DRV: Dietary Reference Value, according to [S4] for male/females aged >14 years.

^c^ 1 Units of vitamin A: equivalents from retinoids (0.3 μg of retinol), and carotenoids (1.8 μg of beta-carotene).

# Table S6. Lipid Profile of Raw Chickpeas.

| **Lipid** | **Value (g) ^a^** | **Content (%) ^b^** | **Content (%) ^c^** | **Daily intake ^d^** |
| --- | --- | --- | --- | --- |
| Total, monounsaturated fatty acids | 1.80 | 28.57 | 19 | 30-40 |
| Total, poliunsaturated fatty acids | 2.81 | 44.63 | 66 | 10-20 |
| Total, saturated fatty acids | 0.43 | 6.78 | 15 | As low as possible |
| Miristic acid (14:0) | 0.01 | 0.15 |  |  |
| Palmitic acid (16:0) | 0.51 | 8.47 | 9.20 – 20.40 |  |
| Estearic acid (18:0) | 0.09 | 1.37 | 1.30 – 1.70 |  |
| Oleic acid (18:1, n-9 cis, ω-9) | 1.36 | 21.67 | 21.60 – 22.20 |  |
| Linoleic acid (LA) (18:2, ω-6) | 2.63 | 41.73 | 43.30 – 56.20 | 4 |
| Linolenic acid (ALA) (18:3, ω-3) | 0.10 | 1.62 | 0.50 – 2.00 | 0.5 |
| Araquidonic acid (20:0) | 0.04 | 0.67 |  |  |
| Eicosadienoic acid (20:2) | 0.03 | 0.46 |  | 250 mg ^e^ |
| Erucic acid (22:1) | 0.02 | 0.33 |  |  |

^a^ Values in g per 100 grams of dry product [S3].

^b^ The values are expressed as % of fat (6.30 g/ 100 g of dry basics) [S3].

^c^ Values expressed as % of fat [6, S5, S6].

^d^ Daily nutritional requirements are based on a caloric intake of 2000 kcal for adults [S4], and expressed as % of total energy intake.

^e^ Eicosapentanoic (EPA) + Docosahexaenoic Acid (DHA).

# Table S7. Amino acid profile of Raw Chickpea Flour.

| **Amino acid** | **mg/g protein ^a^** | **mg/g protein ^b^** | **mg/g protein ^c^** | **Daily intake ^d^**  **WHO/FAO/UNU (2007)** |
| --- | --- | --- | --- | --- |
| Asp ± Asn, D | 114.36 ± 1.35 | 115 – 135 | 112 – 129 |  |
| Glu ± Gln, E | 164.63 ± 1.52 | 178 – 187 | 131 – 175 |  |
| Ser, S | 54.29 ± 0.49 | 37 – 59 | 52 – 67 |  |
| Thr, T | 36.52 ± 0.57 | 30 – 41 | 33 – 51 | 23 |
| Cys, C | 9.60 ± 0.49 | 6 – 11 | 8 – 20 | 6 |
| His, H | 25.93 ± 0.29 | 23 – 32 | 17 – 24 | 15 |
| Arg, R | 98.24 ± 2.59 | 85 – 104 | 83 – 137 |  |
| Lys, K | 66.72 ± 0.90 | 70 – 72 | 49 – 67 | 45 |
| Gly, G | 37.51 ± 0.41 | 36 – 43 | 32 – 45 |  |
| Ala, A | 41.49 ± 0.53 | 46 – 52 | 35 – 47 |  |
| Pro, P | 0.29 ± 0.00 | 18 – 41 | 38 – 65 |  |
| Val, V | 42.63 ± 0.82 | 41 – 46 | 29 – 46 | 39 |
| Met, M | 6.52 ± 0.53 | 8 – 11 | – | 16 |
| Ile, I | 40.58 ± 0.78 | 36 – 48 | 26 – 39 | 30 |
| Leu, L | 72.59 ± 0.86 | 80 – 85 | 56 – 72 | 59 |
| Tyr, Y | 26.80 ± 1.56 | 23 – 28 | 22 – 33 |  |
| Trp, W | 8.70 ± 0.00 | 8 – 9 | 7 – 16 | 6 |
| Phe, F | 57.82 ± 0.74 | 53 – 60 | – |  |
| Met + Cys | 16.09 ± 0.78 | 17 – 19 | – | 22 |
| Phe + Tyr | 84.61 ± 2.22 | 81 – 83 | – | 38 |

The values are presented in mg per gram of dry product according to:

^a^ [39].

^b^ [6].

^c^ [S7].

^d^ Essential amino acid adults scoring pattern requirements (mg/g protein) [S8], considering a mean of nitrogen requirement of 105 mg nitrogen/kg body weight per day (0.66 g protein/kg body weight per day).

# Supplementary References

S1. Page MJ, McKenzie JE, Bossuyt PM, et al (2021) The PRISMA 2020 statement: an updated guideline for reporting systematic reviews. BMJ 372. https://doi.org/10.1136/bmj.n71

S2. Kou X, Gao J, Xue Z, Zhang Z, Wang H, Wang X (2013) Purification and identification of antioxidant peptides from chickpea (*Cicer arietinum* L.) albumin hydrolysates. LWT Food Sci Technol 50:591–598. https://doi.org/10.1016/j.lwt.2012.08.002

S3. AESAN/BEDCA (2010) Base de Datos Española de Composición de Alimentos v1.0. https://www.bedca.net/bdpub/. Accessed 20 February 2024

S4. Alonso Calleja C, Cámara Hurtado M, Daschner A et al (2019) Report of the Scientific Committee of the Spanish Agency for Food Safety and Nutrition (AESAN) on the Nutritional Reference Intakes for the Spanish population. Revista del Comité Científico de la AESAN 29:43–68

S5. Chavan JK, Kadam SS, Salunkhe DK, Beuchat LR (1987) Biochemistry and technology of chickpea (*Cicer arietinum* L.) seeds. Crit Rev Food Sci Nutr 25:107–158. https://doi.org/10.1080/10408398709527449

S6. Rachwa-Rosiak D, Nebesny E, Budryn G (2015) Chickpeas—composition, nutritional value, health benefits, application to bread and snacks: a review. Crit Rev Food Sci Nutr 55:1137–1145. https://doi.org/10.1080/10408398.2012.687418

S7. Jukanti AK, Gaur PM, Gowda CLL, Chibbar RN (2012) Nutritional quality and health benefits of chickpea (*Cicer arietinum* L.): a review. Br J Nutr 108:S11–S26. https://doi.org/10.1017/S0007114512000797

S8. Joint WHO/FAO/UNU Expert Consultation (2007) Protein and amino acid requirements in human nutrition. World Health Organ Tech Rep Ser 935:1–265.

# Supplementary References included in the manuscript as RS

RS1. Cutroneo S, Petrusan JI, Stolzenberger R, Zurlini C, Tedeschi T (2024) Formulation of new sourdough bread prototypes fortified with non-compliant chickpea and pea residues. Front Nutr 11:1351443. https://doi.org/10.3389/fnut.2024.1351443

RS2. Tawalbeh D, Ahmad F, Alu’datt MH, Mhd Sarbon N (2025) Production improvement of Kabuli chickpea (*Cicer arietinum* L.) protein hydrolysates through ultrasonic pre-treatment approach: Impact on techno-functional properties and antioxidant activity. Food Chem Adv 9:101175. https://doi.org/10.1016/j.focha.2025.101175

RS3. Tawalbeh D, Ahmad F, Sengupta S et al (2025) Purification, characterization, and toxicity of antioxidative peptides from ultrasonicated chickpea (*Cicer arietinum* L.) protein hydrolysate. Process Biochem 154:220–233. https://doi.org/10.1016/j.procbio.2025.04.026

RS4. Tawalbeh D, Alu’datt MH, Wan Ahmad WAN et al (2025) Investigating the impact of ultrasound-assisted extraction on the angiotensin-converting enzyme (ACE) inhibitory activity of chickpea (*Cicer arietinum* L.) peptides: *In vitro*, in silico, and acute toxicity study. Bioorg Chem 166:109130. https://doi.org/10.1016/j.bioorg.2025.109130

RS5. Figueroa-Salcido OG, Arámburo-Gálvez JG, Flores-Mendoza LK et al (2025) An optimized chickpea protein hydrolysate exerts long-term antihypertensive effects and upregulates ACE2 and Mas1 gene expression in spontaneously hypertensive rats. Foods 14:3537. https://doi.org/10.3390/foods14203537

RS6. Mahgoub SA, Almutairi HH, Abd-El Fattah NHI et al (2025) Isolation and characterization of novel bioactive peptides from legume seed wastes and their application as eco-friendly coating improve the physicochemical, microbiological, aroma, and ripening properties in probiotic-enriched Ras cheese. Results Eng 26:105352. https://doi.org/10.1016/j.rineng.2025.105352

RS7. Navarro-Leyva A, López-Angulo G, Delgado-Vargas F et al (2026) Antidiabetic activity of chickpea albumin hydrolysate in streptozotocin-induced diabetic rats. J Food Sci 91. https://doi.org/10.1111/1750-3841.71178

RS8. Gharaviri M, Aleksanochkin DI, Ahangaran M et al (2026) Chickpea protein hydrolysates: Production, bioactivity, functional profile, and technological properties. Foods Raw Mater 14:198–213. https://doi.org/10.21603/2308-4057-2026-1-666

RS9. Garza-Aguilar SM, Ceballos-Rubio CD, Serrano-Sandoval SN, Antunes-Ricardo M (2026) Unlocking flavor from legumes: chickpea protein hydrolysates as next-generation flavor enhancers. Future Foods 14:101135. https://doi.org/10.1016/j.fufo.2026.101135

RS10. Borim LC, Dias SCO, Lucio TF, Silva BCG, Moura AT, Coelho RL, Pacheco MTB, Tavano OL (2026) Globulins from a new Brazilian chickpea cultivar GB Cappuccino: insights into compositional, digestibility, and bio-functional potential of their hydrolysates. Macromol 6:11. https://doi.org/10.3390/macromol6010011
